# Identifying individual risk rare variants using protein structure guided local tests (POINT)

**DOI:** 10.1371/journal.pcbi.1006722

**Published:** 2019-02-19

**Authors:** Rachel Marceau West, Wenbin Lu, Daniel M. Rotroff, Melaine A. Kuenemann, Sheng-Mao Chang, Michael C. Wu, Michael J. Wagner, John B. Buse, Alison A. Motsinger-Reif, Denis Fourches, Jung-Ying Tzeng

**Affiliations:** 1 Department of Statistics, North Carolina State University, Raleigh, North Carolina, United States of America; 2 Department of Quantitative Health Sciences, Lerner Research Institute, Cleveland Clinic, Cleveland, Ohio, United States of America; 3 Bioinformatics Research Center, North Carolina State University, Raleigh, North Carolina, United States of America; 4 Department of Statistics, National Cheng-Kung University, Tainan, Taiwan; 5 Public Health Sciences Division, Fred Hutchinson Cancer Research Center, Seattle, Washington, United States of America; 6 Center for Pharmacogenomics and Individualized Therapy, University of North Carolina, Chapel Hill, North Carolina, United States of America; 7 Department of Medicine, University of North Carolina School of Medicine, Chapel Hill, North Carolina, United States of America; 8 Department of Chemistry, North Carolina State University, Raleigh, North Carolina, United States of America; 9 Institute of Epidemiology and Preventive Medicine, National Taiwan University, Taipei, Taiwan; Koç University, TURKEY

## Abstract

Rare variants are of increasing interest to genetic association studies because of their etiological contributions to human complex diseases. Due to the rarity of the mutant events, rare variants are routinely analyzed on an aggregate level. While aggregation analyses improve the detection of global-level signal, they are not able to pinpoint causal variants within a variant set. To perform inference on a localized level, additional information, e.g., biological annotation, is often needed to boost the information content of a rare variant. Following the observation that important variants are likely to cluster together on functional domains, we propose a protein structure guided local test (POINT) to provide variant-specific association information using structure-guided aggregation of signal. Constructed under a kernel machine framework, POINT performs local association testing by borrowing information from neighboring variants in the 3-dimensional protein space in a data-adaptive fashion. Besides merely providing a list of promising variants, POINT assigns each variant a p-value to permit variant ranking and prioritization. We assess the selection performance of POINT using simulations and illustrate how it can be used to prioritize individual rare variants in *PCSK9, ANGPTL4* and *CETP* in the Action to Control Cardiovascular Risk in Diabetes (ACCORD) clinical trial data.

## Author summary

While it is known that rare variants play an important role in understanding associations between genotype and complex diseases, pinpointing individual rare variants likely to be responsible for association is still a daunting task. Due to their low frequency in the population and reduced signal, localizing causal rare variants often requires additional information, such as type of DNA change or location of variant along the sequence, to be incorporated in a biologically meaningful fashion that does not overpower the genotype data. In this paper, we use the observation that important variants tend to cluster together on functional domains to propose a new approach for prioritizing rare variants: the protein structure guided local test (POINT). POINT uses a gene’s 3-dimensional protein folding structure to guide aggregation of information from neighboring variants in the protein in a robust manner. We show how POINT improves selection performance over existing methods. We further illustrate how it can be used to prioritize individual rare variants using the Action to Control Cardiovascular Risk in Diabetes (ACCORD) clinical trial data, finding promising variants within genes in association with lipoprotein-related outcomes.

## Introduction

Rare genetic variants, e.g. those which occur in less than 1-3% of a population, play an important role in complex diseases. Individual rare variants can be difficult to detect due to low frequencies of the mutant alleles. Therefore, associations involving rare variants are typically discerned using “global” or variant-set tests, which aggregate information across variants to gain sufficient power. These aggregation tests can be done in a burden-based fashion (i.e., modeling phenotype as a function of a weighted sum of genetic markers) [1–4], or using kernel tests (i.e., examining association between pairwise trait similarity and pairwise genetic similarity) [5–9]. Global aggregation tests substantially improve the power for detecting set-level association with phenotypes; however, they are not able to identify individual rare risk variants responsible for the set-level significance.

Localizing rare risk variants from a significant variant set can help guide follow-up studies and provide insight into the functionality and molecular mechanisms of the phenotypes. Several methods have been proposed to prioritize individual rare risk variants based on single-variant analysis [10–12]; yet it has been shown that borrowing external information, either from biological annotations or from other rare variants, can amplify the information content, better separate causal and non-causal variants, and significantly stabilize inferences made at the local level [13].

One approach for variant prioritization involves using functional annotation to filter out variants that are less likely to be causal [14, 15]. Informative functional annotation may include variant frequency, type of DNA change (e.g., frameshift, missense, etc.), conservation score, and predicted impact of the variant on protein structure and gene constraint [15]. While useful for providing a subset of likely causal variants, annotation-based filtering is often phenotype non-specific, and may lead to high false negative selection rates when rigid variant-exclusion thresholds are applied based on one or more filtering criteria [15].

A second class of prioritization methods incorporates functional information as a prior to avoid using absolute rules to include or exclude variants. These functional priors, typically imposed on variant effects, have been included in hierarchical modeling frameworks [13, 16, 17] and Bayesian variable selection models [18, 19]. Methods of this type reduce the occurrence of false negatives as described above and allow the trait-variant association to guide variant selection, yielding better prioritization performance. In addition, these hierarchical approaches facilitate estimation of individual effects of the rare variants. However, these methods can be computationally demanding as the computational burden grows with increasing numbers of variants.

A third class of prioritization methods searches for genomic clustering of rare risk variants. These methods stem from the observation that functional or disease-causing variants are more likely to cluster together than null variants [20–23] in the functional domains. Yue et al. [20] note the existence of “domain hotspots”, or mutational hotspots, within which known functionally significant mutations are more likely to cluster together compared to random nonsynonymous variants. Frank et al. [21] discuss significant clusters of variants within glutamate domains in schizophrenia and bipolar disorder. It has also been shown that actions and interactions of regulatory elements (e.g., promoters, repressors, and enhancers) may be one key reason for relevant loci to cluster within functional domain or mutational hotspots [22]. Based on the observance of domain hotspots, various methods have been proposed to exhaustively search for the single nucleotide polymorphism (SNP) subset that is most significantly associated with the phenotype, either in 2-dimensional (2D) sequence space [24–27] or among all possible SNP subsets [28–32]. All-subset searches may provide better coverage, especially when risk variants do not cluster closely together in the 2D sequence space (such as in the case of regulatory elements). However, the computational burden of an all-subset search can be intractable when a large number of variants are of interest, and consequently require splitting up the target genomic region into segments beforehand [29], which may lead to missing an optimal subset split over arbitrarily defined segments.

In this work, we propose the **p**r**o**te**in** structure guided local **t**est (POINT) as a new method for prioritizing individual risk rare variants. Like the third class of prioritization methods which focuses on genomic clusters to pinpoint rare causal variants, POINT is built upon the rationale that risk variants tend to cluster within functional domains or mutational hotspots [20–23]. In order to search beyond the 2D sequence space yet retain computational efficiency, however, POINT relies on the tertiary protein structure, i.e., the 3-dimensional (3D) folding of amino acids, to guide local collapsing from nearby variants in the functional domain.

Specifically, POINT incorporates the 3D protein structure into the kernel machine regression framework, defining a local kernel function to enable variant-specific information collapsing. For a given variant, the amount of information contributed from its neighboring variants decays with the distance between variants in the 3D protein space. POINT performs local score tests for each variant over a range of kernel scale values, adaptively choosing the maximum distance allowed for information collapsing. In particular, for each variant, POINT calculates the minimum p-value (minP) across different distances, and uses a resampling approach to compute the p-value of minP, which can then be used to rank and select promising variants.

Below we evaluate the prioritization performance of POINT using simulation studies. We also apply POINT to the Action to Control Cardiovascular Risk in Diabetes (ACCORD) clinical trial data, finding promising rare variants in *PCSK9*, *ANGPTL4* and *CETP* that may be associated with lipoprotein-related outcomes.

## Methods

### Overview of POINT

We consider a study of *n* subjects with phenotype *Y*_*n*×1_ = [*Y*_1_, …, *Y*_*n*_]^*T*^. We assume *Y* follows an exponential family distribution with canonical link *g*(*μ*) = *g*(*E*[*Y*|*X*, *G*]), where *G*_*n*×*M*_ is the genotype design matrix of the *M* variants, and *X*_*n*×*p*_ is a matrix of the *p* non-genetic covariates. A kernel machine (KM) model for the local effect of variant *m*, *m* = 1, …, *M*, is of the form

$$\begin{array}{c} g\left(\mu \right)=X\beta +h^{m}\left(G\right) \end{array}$$

where $h^{m}\left(G\right)\equiv h^{m}=\sum_{j=1}^{n}\alpha_{j}^{m}k\left(G,g_{j}\right)$ is a *n* × 1 vector of the effect of variant *m*, and $g_{j}^{T}=\left[g_{j1},...,g_{jM}\right]$ is the *j*th row of *G* and is genotype design vector for individual *j*. We assume *h*^*m*^ ∼ *N*(0, *τ*_*m*_ *K*^*m*^), where the *n* × *n* matrix *K*^*m*^ = {*k*^*m*^(*g*_*i*_, *g*_*j*_)} is a local kernel matrix for variant *m*, describing the covariance between the local effect of variant *m* for different individuals. The local kernel matrix *K*^*m*^ is constructed in a manner such that *K*^*m*^ only puts non-trivial weights on the genetic similarity from variants that are in close proximity to variant *m*, with closer neighboring variants receiving higher weights. As detailed later, the local kernel function *k*^*m*^(*g*_*i*_, *g*_*j*_) uses the distance between variants in the 3D protein space to determine the amount of contribution from neighboring variants when quantifying the localized genetic similarity about variant *m*. From the local kernel, we construct a local kernel test with null hypothesis *H*_0_: *τ*_*m*_ = 0 to evaluate if variant *m*, along with its proximal neighboring variants, are associated with the phenotype.

**Fig 1:**
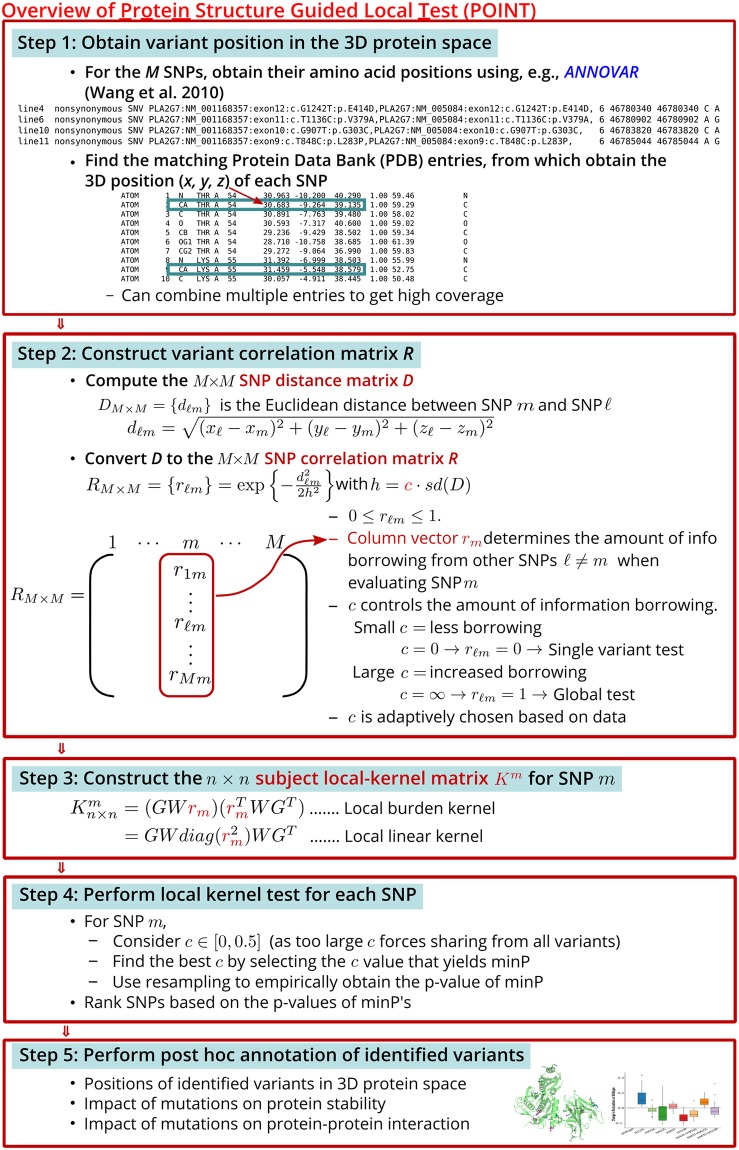
Overview of the protein structure guided local test (POINT).

POINT consists of five main steps: (1) obtain the position of each variant in the 3D protein space, (2) construct a variant correlation matrix using the Euclidean distance between variants in the 3D protein space, (3) construct protein structure guided kernel matrices, (4) perform a local kernel test of *H*_0_: *τ*_*m*_ = 0 for variant *m* over a range of collapsing distances and obtain the p-value, and finally (5) perform post hoc annotations of identified variants. The workflow is illustrated in Fig 1. Each step is further described below.

#### Step 1: Obtaining variant positions in the 3D protein space

The first step in performing the protein structure guided local test is to collect protein tertiary structure information, in the form of 3D coordinates in the protein space, for each of the *M* variants of interest. In order to do so, we must first map our genotype data to an appropriate Protein Data Bank (PDB) [33] entry. Based on the variant position on the DNA sequence, one can use the annotation tool ANNOVAR [34] to obtain the gene name, DNA mutation, and corresponding amino acid position and mutation of each SNP of interest. These amino acid mutations can be manually aligned to PDB entries for the gene of interest.

When a gene has multiple protein structure entries available on PDB, we select an appropriate entry for our analysis. Optimal PDB structures should have high resolution (≤ 2.0Å), good data quality (e.g., low percent outliers, clashscore and Rfree score), and high coverage of variant 3D protein position for our variant set. Once an appropriate PDB entry has been identified, we extract the 3D coordinates (*x*, *y*, *z*) for each variant of interest, either using the coordinates of the carbon alpha for that particular amino acid residue, or taking the average of the coordinates of all the atoms forming the side chain of that residue (also called side chain centroid).

#### Step 2: Constructing variant correlation matrix *R*

From the 3D Cartesian coordinates obtained in Step 1, we build a SNP pairwise distance matrix, *D*_*M*×*M*_ = {*d*_*ℓm*_}, where *d*_*ℓm*_ = [(*x*_*ℓ*_ − *x*_*m*_)^2^ + (*y*_*ℓ*_ − *y*_*m*_)^2^ + (*z*_*ℓ*_ − *z*_*m*_)^2^]^1/2^ is the Euclidean distance between variants *ℓ* and *m* on the protein tertiary structure, and *d*_*ℓm*_ = 0 if *ℓ* = *m*. Using distance matrix *D*, we form a *M* × *M* variant correlation matrix $R=\left\{r_{\ell m}\right\}=\exp\{-\frac{d_{\ell m}^{2}}{2h^{2}}\}$, 0 ≤ *r*_*ℓm*_ ≤ 1.

Although we call *R* the variant correlation matrix, rather than inducing correlation between variants, matrix *R* is used to induce smoothing of information between neighbors, allowing gradual drop-off in the amount of borrowing between variants as the variant distance increases. Specifically, let *r*_*m*_ be the *m*^*th*^ column vector of *R* with dimension *M* × 1; when testing for variant *m*, *r*_*m*_ determines the relative contribution from each other variant *ℓ* = 1, …, *M* (noting that *r*_*mm*_ = 1) via scale parameter *h*.

Rather than performing parameter tuning to determine an optimal scale *h*, we follow a similar approach to Tango [35] and Schaid et al. [27] and examine a grid of values. However, to make *h* scale free, instead of using proportion of maximum distance as a metric, we consider a grid over the proportion of the standard deviation of all pairwise Euclidean distances between variants, i.e., *h* = *c* ⋅ *sd*(*D*). The idea behind using a function of the standard deviation of pairwise distances is borrowed from nonparametric theory for choosing the optimal bandwidth of kernels.

Expressed in this manner, parameter *c* is used as a proxy for separating variants, so that only those variants within the “neighborhood” (or cluster) of variant *m* on the protein structure are likely to contribute information when quantifying localized genetic similarity around variant *m*. Larger *c* values encourage information contributed from a larger neighborhood, and the local test becomes a global-level test (i.e., *r*_*ℓm*_ = 1 for all *ℓ*) when *c* = *∞*. Smaller *c* values allow information to be contributed from a smaller neighborhood, and the local test becomes a single-variant test (i.e., *r*_*mm*_ = 1 and *r*_*ℓm*_ = 0 for *ℓ* ≠ *m*) when *c* = 0.

When conducting local kernel tests in Step 4, we consider a grid of *c* values between 0 and 0.5 and let the data adaptively choose the best scale *c*. This strategy provides two layers of protection to safeguard against false positive selections. First, as illustrated later in Table 1 of simulation results, setting *c* = 0.5 as the maximum proportion of standard deviation of distance allowed for borrowing forces information sharing only from variants within a localized neighborhood in the protein tertiary space. In contrast, a larger maximum *c* value would result in information sharing from variants across the protein, regardless of whether they are close enough to be expected to share biological architecture or not. Consequently, a larger maximum *c* may lead to higher chances of selecting non-causal variants as promising loci. The second layer of protection stems from the fact that with the adaptively determined *c*, structure is used as a prior where, rather than forcing sharing of information between variants which may not have related genetic effects on phenotype, neighboring information can be shared only if there appears to be sufficient support from the data to do so.

**Table 1:** Counts of neighboring variants which contribute “significantly” to the focal rare variants in *PLA2G7* for different values of c. Neighboring variants *ℓ*’s contributing ≥ 5% of the information to the focal variant *m* (i.e., neighboring variants with *r*_*ℓm*_ ≥ 0.05) are considered as “significant”.

Focal variant	Neighboring variants within cluster*	Number of neighboring variants with *r*_*ℓm*_ ≥ 0.05 within the neighborhood defined by *c*										
		0.1	0.2	0.3	0.4	0.5	0.6	0.7	1	1.5	2	4
D69	R82	0	1	1	1	1	2	3	9	12	12	12
R82	D69	0	1	1	1	1	2	2	9	12	12	12
F110	S273	0	0	0	0	1	3	3	11	12	12	12
D181	T187	0	0	0	1	1	4	5	12	12	12	12
T187	D181	0	0	0	1	1	6	7	9	12	12	12
K191	D200	0	0	0	1	1	2	2	6	10	12	12
D200	K191	0	0	0	1	1	2	3	6	9	12	12
S273	F110	0	0	0	0	1	5	7	10	12	12	12
V279	L283	0	0	1	1	2	5	5	10	12	12	12
L283	V279	0	0	1	1	1	3	3	9	12	12	12
G303	A326, M331	0	0	0	0	1	4	6	7	11	12	12
A326	G303, M331	0	0	0	1	1	3	3	7	10	12	12
M331	G303, A326	0	0	0	1	3	5	5	7	10	12	12

**Fig 2:**
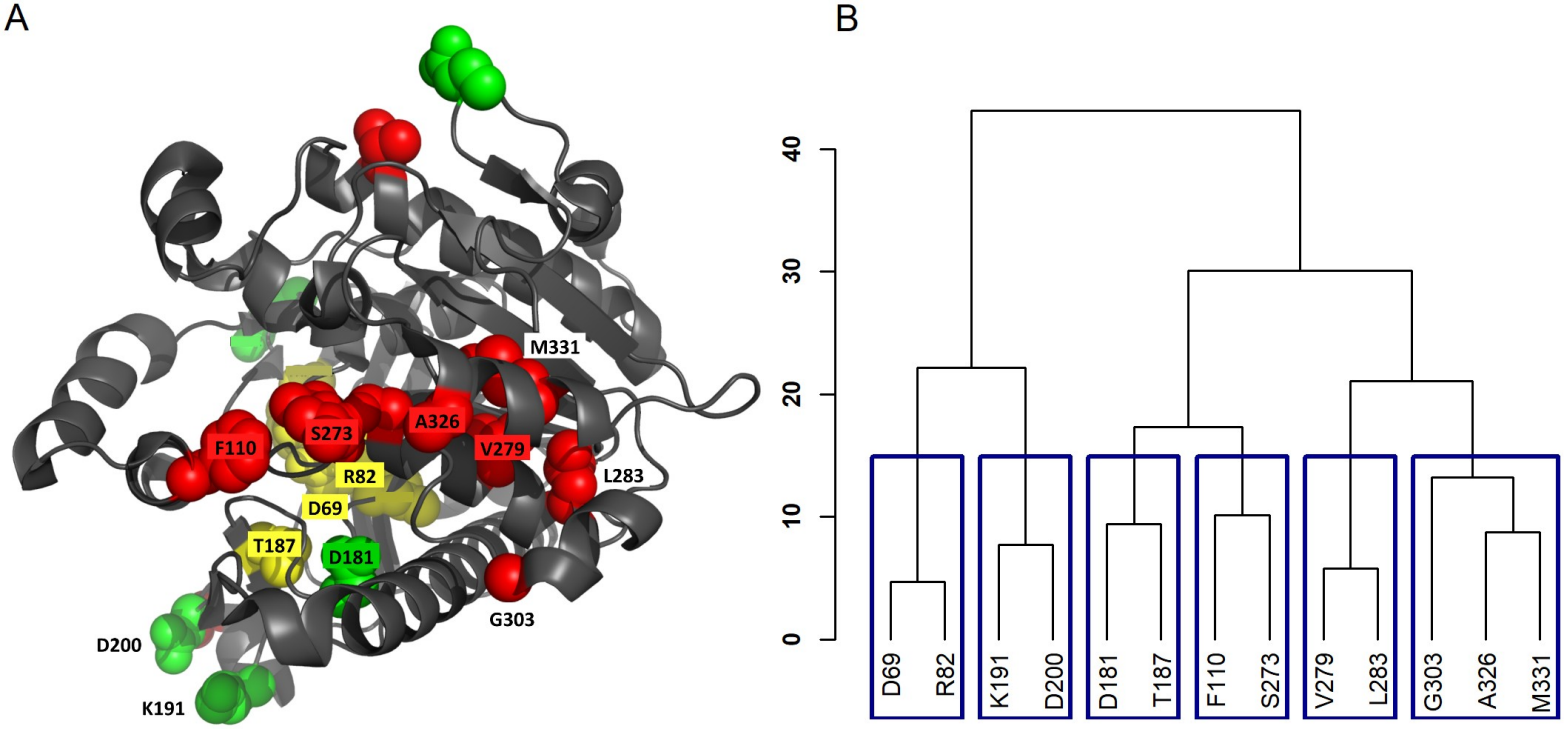
*PLA2G7* rare variant positions. A: Rare variant locations on the protein tertiary structure. B: Corresponding Euclidean distance-based clustering of the variants.

*: The cluster is defined based on the variants’ position in the 3D space as shown in Fig 2.

#### Step 3: Constructing local subject kernel matrix *K*^*m*^ for variant *m*

Given the *M* × *M* variant correlation matrix *R*, we create the *n* × *n* subject kernel matrix *K*^*m*^ that quantifies the genetic similarity between all pairs of individuals at variant *m* and its neighboring variants. By incorporating information from *R* as an additional weight, the commonly used global kernel functions can be extended to local kernels, where genetic similarity is calculated based largely on focal variant *m* and less on neighboring variants, with effect decaying with distance. To illustrate, let *w_ℓ_* be the variant-specific weight for variant *ℓ* (e.g., based on the MAF or functional impact of variant *ℓ*) and let *r*_*ℓm*_ be the (*ℓ*, *m*)^*th*^ entry of the variant correlation matrix *R*. To quantify the similarity between subjects *i* and *j*, the global burden kernel function is $k^{global}\left(g_{i},g_{j}\right)=(\sum_{\ell =1}^{M}w_{\ell }g_{i\ell })(\sum_{\ell =1}^{M}w_{\ell }g_{j\ell })$ [36]. The local burden kernel function can be obtained as $k^{m}\left(g_{i},g_{j}\right)=(\sum_{\ell =1}^{M}r_{\ell m}w_{\ell }g_{i\ell })(\sum_{\ell =1}^{M}r_{\ell m}w_{\ell }g_{j\ell })$. The additional weight *r*_*ℓm*_ in the local kernel function controls the amount of contribution from variant *ℓ*, which diminishes as variant *ℓ*’s distance from the focal variant *m* increases. Following this idea and using a matrix representation, we have the local burden kernel as $K^{m}=\left(GWr_{m}\right)\left(r_{m}^{T}WG^{T}\right)$ where *W* = *diag*(*w*_1_, …, *w*_*M*_), the linear local kernel as $K^{m}=GW\text{diag}\left(r_{m}^{2}\right)WG^{T}$, and the polynomial local kernel as $K^{m}={\left(1+GW\text{diag}\left(r_{m}^{2}\right)WG^{T}\right)}^{d}$. When *r*_*m*_ = **1**_*M*×1_, the local kernel matrix becomes the global kernel matrix.

#### Step 4: Performing local kernel test of *H*_0_: *τ*_*m*_ = 0 for variant *m*

The local test of *H*_0_: *τ*_*m*_ = 0 assesses whether variant *m*, along with its nearby variants (within a small neighborhood defined by *c*) are associated with the phenotype. To describe the score-based test statistic of the local test, we further rewrite the kernel matrix *K*^*m*^ as *K*^*m*,*c*^ to emphasize that it is computed at a fixed value of *c*. Following Wu et al. [8] and Tzeng et al. [37, 38], the score-based test statistic *T*_*m*,*c*_ has a quadratic form and follows a weighted chi-square distribution asymptotically. Specifically, $T_{m,c}=\frac{1}{n}{\left({\hat{\epsilon }}_{1},...,{\hat{\epsilon }}_{n}\right)}^{T}K^{m,c}\left({\hat{\epsilon }}_{1},...,{\hat{\epsilon }}_{n}\right)$, where ${\hat{\epsilon }}_{i}$ is the fitted residual for the KM model under the null hypothesis, i.e., ${\hat{\epsilon }}_{i}=Y_{i}-g^{-1}(X_{i}^{T}\hat{\beta })$ where $X_{i}^{T}$ is the 1 × *p* row vector of the covariate design matrix *X*. The weights of the weighted chi-square distribution are given in S1 Appendix. Therefore, for a fixed *c*, the corresponding p-value, denoted by *p*_*m*,*c*_, can be calculated using the Davies method [39].

Given a grid of *c*’s, *c* = *c*_1_, …, *c*_*L*_, we adaptively find the optimal *c* by choosing the value that yields the minimum *p*-value for variant *m*, i.e., $\text{minP}=\text{min}\{p_{m,c_{1}},...,p_{m,c_{L}}\}$. As shown in S1 Appendix, we develop a resampling approach to calculate the p-value of the minP statistic, denoted by $p_{m}^{*}$. These p-values can be used to rank and select promising variants, e.g., to select the top variants whose p-value $p_{m}^{*}$ is less than a certain threshold.

#### Step 5: Performing post hoc annotations of identified variants

Once the promising variants are identified, we examine the functional roles and potential structural consequences of these mutations. The post-hoc analysis can include the examination of the variant locations on the protein three-dimensional structure, searches in annotation literature and databases (e.g., the Universal Protein Resource (UniProt)) to understand the potential biological mechanisms, and even the quantitative evaluation of conformational changes of the mutant protein (e.g., via molecular dynamic simulations (MDS)) to predict how the identified mutations could affect the protein’s stability and interactions with other proteins.

### Simulation study set-up

We design a simulation following the work of Song et al. [23], which examined the effect of SNPs within Phospholipase A2 Group VII (*PLA2G7*) on protein function and enzyme activity of Lipoprotein-associated phospholipase A_2_ (Lp-PLA2) measured on ∼90 individuals. Genotype data from Sanger sequencing of *PLA2G7* are also available on 2000 individuals from the CoLaus study, a study examining psychiatric, cardiovascular, and metabolic disorders in 6188 Caucasians aged 35-75 from Lausanne, Switzerland [40, 41]. Song et al. [23] found that variants which are deemed likely to be non-null variants for enzyme activity of Lp-PLA2 tend to cluster together and are predominately on the surface of protein, while null variants are nearby in the core of protein [23]. For the simulation study, we obtain the sequencing genotypes of *PLA2G7* from Song et al. [23] and obtain the variants’ 3D coordinates on the protein tertiary structure from PDB entry 3F96 [42]. In total, 13 rare variants from the Song et al. [23] study have protein coordinate information available in PDB; their variant information is provided in S1 Table. Fig 2 shows the variants’ location in the 3D protein structure and the corresponding Euclidean distance-based clustering of these variants. In the figure, each variant is named by its amino acid position on the 2D sequence structure.

Using the genotype data from these 13 variants, we generate phenotypes for *n* individuals, with *n* = 1000 or 2000, from the model of *g*(*μ*) = *β*_0_ + *G**β***_*G*_; we use identity link *g*(*μ*) = *μ* for continuous traits (i.e., $y_{i}\overset{iid}{∼}N\left(\mu_{i},\sigma =1\right)$) and use logit link *g*(*μ*) = exp(*μ*)/(1 + exp(*μ*)) for binary traits. We set the intercept *β*_0_ = 0.5 for continuous traits, and *β*_0_ = −0.05 for binary traits, and set the coefficient vector ***β***_*G*_ = {*β*_*G*,*m*_} of genetic effects as *β*_*G*,*m*_ = *b* × |log_10_(*MAF*_*m*_)|, where *b* ≠ 0 for causal variants and is equal to zero otherwise, and *MAF*_*m*_ is the minor allele frequency of variant *m*. This specification of *β*_*G*,*m*_ assigns larger effects to rarer variants.

We consider a variety of scenarios for causal variants: Scenario (A): One cluster is causal, where the causal variants cluster close together on the tertiary protein structure, with varying closeness on the amino acid sequence; we consider four sub-scenarios with (D69, R82), (F110, S273), (K191, D200), and (G303, A326, M331), chosen to be the causal variant clusters. Scenario (B): Part of a cluster is causal, where only a subset of closely clustered variants are causal; we consider four sub-scenarios with (D69), (F110), (K191), (A326, M331) respectively from the variant clusters in Scenario (A) are causal. Scenario (C): Two opposing clusters are causal, where two clusters of variants are causal, with one cluster, (D69, R82), positively conferring phenotype risk, and another cluster, (G303, A326, M331), negatively conferring phenotype risk. These chosen causal variants also cover a range of causal variant frequencies, from 0.0039 to 0.0200; detailed information can be found in Tables 2 and 3. Finally, we also consider the scenario of no causal variants to examine the validity of the proposed POINT tests.

**Table 2:** Selection performance of continuous-trait simulation with *n* = 2000 subjects. Selection performance for single variant test (SVT), REBET, POINT test using local burden kernel (POINT-Burden), and POINT test using local linear kernel (POINT-Linear). The best performed methods (based on the composite F-measure) are shown in bold and the second best are shown in *italic*.

		Scenario (A) One Causal Cluster				Scenario (B) Part of Cluster is Causal				Scenario (C) Two Opposing Causal Clusters
causal variants		(D69, R82)	(F110, S273)	(K191, D200)	(G303, A326, M331)	D69	F110	K191	(A326, M331)	(D69, R82), (G303, A326, M331)
MAF*		(0.0050, 0.0200)	(0.0095, 0.0065)	(0.0045, 0.0055)	(0.0385, 0.0095, 0.0085)	0.0050	0.0095	0.0045	(0.0095, 0.0085)	(0.0050, 0.0200), (0.0385, 0.0095, 0.0085)
TPR	SVT	0.665	0.569	0.563	0.687	0.528	0.724	0.546	0.648	0.751
	REBET	0.752	0.175	0.430	0.984	0.072	0.172	0.108	0.296	0.947
	POINT-Burden	0.910	0.695	0.740	0.826	0.456	0.723	0.521	0.797	0.903
	POINT-Linear	0.860	0.605	0.628	0.717	0.480	0.712	0.575	0.695	0.830
FDR	SVT	0.423	0.306	0.313	0.196	0.494	0.431	0.492	0.273	0.209
	REBET	0.459	0.249	0.352	0.209	0.613	0.488	0.561	0.410	0.282
	POINT-Burden	0.364	0.258	0.246	0.176	0.566	0.426	0.519	0.234	0.198
	POINT-Linear	0.394	0.319	0.308	0.222	0.572	0.467	0.520	0.304	0.203
F Measure	SVT	0.618	0.625	0.619	0.741	**0.517**	*0.637*	**0.527**	0.685	0.771
	REBET	0.629	0.284	0.517	**0.877**	0.121	0.258	0.173	0.394	*0.817*
	POINT-Burden	**0.749**	**0.718**	**0.747**	*0.825*	0.444	**0.640**	0.500	**0.781**	**0.850**
	POINT-Linear	*0.712*	*0.641*	*0.658*	0.746	*0.452*	0.610	*0.523*	*0.696*	0.813

* MAF: Minor allele frequency

**Table 3:** Selection performance of binary trait simulation with *n* = 2000 subjects. Selection performance for single variant test (SVT), scan statistic (SCAN), ADA, REBET, POINT test using local burden kernel (POINT-Burden), and POINT test using local linear kernel (POINT-Linear). The best performed methods (based on the composite F-measure) are shown in bold and the second best are shown in *italic*.

		Scenario (A) One Causal Cluster				Scenario (B) Part of Cluster is Causal				Scenario (C) Two Opposing Causal Clusters
causal variants		(D69, R82)	(F110, S273)	(K191, D200)	(G303, A326, M331)	D69	F110	K191	(A326, M331)	(D69, R82), (G303, A326, M331)
MAF*		(0.0050, 0.0200)	(0.0095, 0.0065)	(0.0045, 0.0055)	(0.0385, 0.0095, 0.0085)	0.0050	0.0095	0.0045	(0.0095, 0.0085)	(0.0050, 0.0200), (0.0385, 0.0095, 0.0085)
TPR	SVT	0.680	0.569	0.560	0.684	0.546	0.708	0.556	0.669	0.723
	SCAN	0.622	0.230	0.518	0.757	0.164	0.338	0.218	0.556	0.399
	ADA	0.627	0.426	0.416	0.609	0.190	0.218	0.182	0.469	0.835
	REBET	0.682	0.151	0.312	0.976	0.062	0.184	0.09	0.298	0.925
	POINT-Burden	0.908	0.677	0.722	0.810	0.468	0.720	0.528	0.787	0.894
	POINT-Linear	0.846	0.620	0.605	0.716	0.489	0.733	0.577	0.673	0.821
FDR	SVT	0.431	0.300	0.299	0.186	0.504	0.425	0.497	0.264	0.218
	SCAN	0.497	0.490	0.339	0.163	0.491	0.486	0.627	0.109	0.735
	ADA	0.650	0.624	0.631	0.479	0.798	0.797	0.807	0.621	0.363
	REBET	0.452	0.237	0.409	0.191	0.587	0.414	0.559	0.369	0.275
	POINT-Burden	0.373	0.269	0.254	0.174	0.571	0.500	0.534	0.240	0.196
	POINT-Linear	0.394	0.314	0.317	0.217	0.569	0.460	0.514	0.298	0.204
F Measure	SVT	0.619	0.628	0.623	0.744	**0.520**	**0.634**	**0.528**	*0.701*	0.751
	SCAN	0.556	0.317	0.581	0.795	0.248	0.408	0.275	0.685	0.319
	ADA	0.450	0.400	0.391	0.562	0.196	0.210	0.187	0.419	0.723
	REBET	0.608	0.252	0.408	**0.885**	0.108	0.280	0.150	0.405	*0.813*
	POINT-Burden	**0.741**	**0.702**	**0.733**	*0.818*	0.448	*0.624*	*0.495*	**0.773**	**0.847**
	POINT-Linear	*0.706*	*0.651*	*0.641*	0.748	*0.458*	0.622	**0.528**	0.687	0.808

* MAF: Minor allele frequency

For POINT, we use weights proportional to a Beta(MAF,1,25) distribution as described in Wu et al. [8] (i.e., *w_ℓ_* = (1 − *MAF_ℓ_*)^24^) to upweight the contribution of rare neighboring variants. We consider a grid of 6 values for *c*, i.e., *c* = (0, 0.1, 0.2, 0.3, 0.4, 0.5) and perform tests using burden and linear kernels, each with 500 replications per scenario, and 1000 resamples per replication. We evaluate the ability of POINT to prioritize causal variants by comparing to the single variant test as well as 3 other methods that also aim to identify the genomic subregions enriched with risk variants. Specifically, the 4 benchmark methods we consider are (i) the single variant score test (which is referred to as SVT and corresponds to POINT with *c* = 0); (ii) the scan statistic of Ionita-Laza et al. [25] (which is referred to as SCAN and has been shown to be the superior method among those searching in 2D space [27]); (iii) ADA of Lin [31] (which identifies important SNPs by searching among all possible subsets of ordered SNPs based on the p-values of single-variant tests); and (iv) REBET of Zhu et al. [32] (which is a subregion-based burden test to identify important SNP subsets among all possible combinations of predefined subgroups within a gene; here subregions are defined based on variants’ biological characteristics or functional domains). In the PLA2G7 simulation, the subregions are defined as: (D69, R82, F110), (D181, T187, K191, D200), (S273, V279, L283) and (G303, A326, M331). SCAN and ADA are only included in the binary case-control simulations as they are only applicable to binary outcomes.

The selection performance of the methods is assessed using true positive rates (TPR), false discovery rates (FDR), and a composite metric called F measure, which is the harmonic mean of the TPR and 1−FDR with 1 being the best and 0 being the worst. TPR is obtained by first computing the fraction of selected causal variants among all causal variants in each replication, and then averaging across the 500 replications. FDR is obtained by first computing the fraction of selected non-causal variants among all selected variants in each replication, and then averaging across the 500 replications. For SVT and POINT, a variant is selected if its p-value is smaller than a pre-specified threshold, e.g., 0.05. For SCAN, a variant is selected if it is included in the best window (i.e., the window with maximum test statistic) and the best window is significant. Similarly, for ADA, a variant is selected if its per-site p-value is less than the optimal threshold (i.e., the p-value threshold yielding the minimum p-value in the observed data) and the overall ADA test p-value is significant. For REBET, a variant is selected if the subregion it falls in is found to be significantly associated from the 2-sided test, which examines both the positively associated and negatively associated subregions. We also evaluate the overall selection performance using empirical receiver operating characteristic (ROC) curves to show the results across all possible decision (e.g., p-value) thresholds.

### Application to the ACCORD study

The ACCORD clinical trial was a multi-center trial with the intent to test for the effectiveness of intensive glycemic, blood pressure, and fenofibrate treatments versus their corresponding standard treatment strategies on cardiovascular disease (CVD) endpoints in subjects with type 2 diabetes [43–46]. The trial enrolled 10,251 subjects with type 2 diabetes and a risk or history of CVD from 77 centers around North America, and found that intensive treatments were not beneficial and were even potentially harmful for some of the CVD endpoints studied [44]. A recent study of this trial investigated genotype associations with individual variation in serum lipid levels in the context of patients with type 2 diabetes [46]. Focusing on the baseline pre-intervention data, Marvel and Rotroff et al. [46] examined the association between baseline blood lipid levels and common variants and rare variants from 16,538 genes in 7,844 ACCORD trial participants that consented to genetic studies. Based on rare variant associations, they found 11 genes to be significantly associated with blood lipid levels, including total cholesterol, low-density lipoprotein (LDL), high-density lipoprotein (HDL), and total triglycerides.

Here we focus on proprotein convertase subtilisin/kexin 9 (*PCSK9*), as it is the gene reported to be most highly associated with LDL from the baseline study of Marvel and Rotroff et al. [46] and of high clinical importance. Because the gene-level rare variant signals in Marvel and Rotroff et al. [46] were mainly identified via burden-based tests, we apply POINT with burden kernels, aiming to prioritize the individual variants associated with LDL within *PCSK9*. Following the work of Marvel and Rotroff et al. [46], we considered rare variants to be those with MAF < 3% and use only individuals with less than 15% missingness. Missing genotype information was imputed previously by Marvel and Rotroff et al. [46]. We use ANNOVAR [34] to find the amino acid position of each variant on the 2D sequence and then obtain the carbon alpha coordinates from PDB entry 4K8R [47], which we determined to be the most representative of the wild type protein while maximizing the number of variants of interest with known protein tertiary position, i.e., 19 of 22 variants. A summary of the 19 variants and the corresponding 3D coordinates from PDB is given in S2 Table. In the analysis, we adjust for 26 baseline covariates as in Marvel and Rotroff et al. [46], including patient age, gender, body mass index (BMI), presence of cardiovascular history, trial treatment arm assignment, top three principal components of ethnic background, years since diabetes and since hyperlipidemia diagnoses, fasting glucose level, and indicators of use of different treatments (e.g., insulin, lipid-lowering drugs, etc.). A full list of these covariates can be found in the Supplementary Materials of Marvel and Rotroff et al. [46].

We compare POINT results with SVT and REBET. For the REBET analysis, we define subregions based on the molecule processing and domain information from UniProtKB (Entry Q8NBP7) and obtain 5 subregions: (R93, R96) for propetide; (N157) for polypeptide chain; (V252∼H417) for peptidase S8 domain; (N425, A443) for variants in-between two domains; and (G466∼R659) for C-terminal domain.

We also repeat the above analysis on those genes that were found to have significant associations in the ACCORD study of Marvel and Rotroff et al. [46] and have accessible information of the 3D protein structure for the genotyped variants in PDB. There are two such genes available: *ANGPTL4* and *CETP*. In *ANGPTL4*, 8 out of the 14 variants have 3D protein structure information in PDB entry 6EUB. In *CETP*, 13 out of the 18 variants have 3D protein structure information in PDB entry 2OBD. For both genes, we aim to identify important variants associated with HDL using SVT, POINT and REBET. The variant information, protein structure, and REBET subregion definitions are shown in S2 Appendix (for *ANGPTL4*) and S3 Appendix (for *CETP*).

## Results

### Results of simulation studies

#### Variant correlation matrix *R* vs. information borrowing from other variants

To illustrate how the variant correlation matrix *R* controls information borrowed from nearby variants, we examine how the range of variants that a focal variant significantly borrows from changes for different values of *c*. For each variant in *PLA2G7*, Table 1 lists its neighboring variant(s) within the same cluster given their position on the 3D protein space (as seen in Fig 2), and the number of variants (excluding the focal variant itself) that “significantly” contribute information to the focal variant for a range of *c* values between 0.1 and 4. Here we use the term “significantly” to loosely indicate those variants *ℓ* whose value *r*_*ℓm*_ is at least 0.05, i.e., contributing at least 5% as much information as the focal variant. We see that for *c* = 0.5, the number of variants that contribute information to the focal variant corresponds well with the true number of variants within the same cluster as it. As *c* increases past 0.5, the number of variants borrowed from for each variant increases dramatically—borrowing from many more than just those that cluster together even for *c* = 0.6 and *c* = 0.7, and borrowing from all variants as *c* increases further.

**Fig 3:**
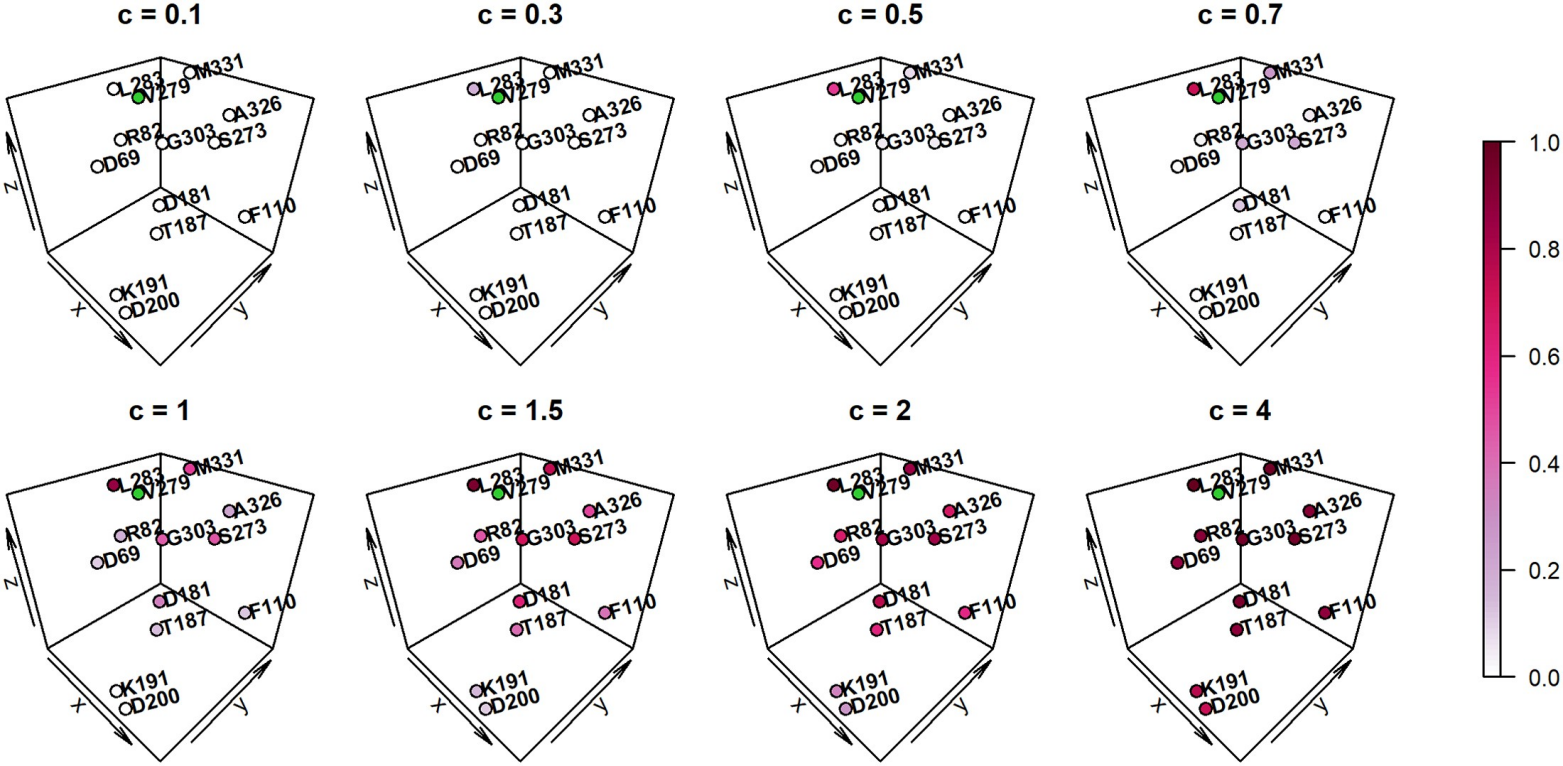
Information-borrowing map for *PLA2G7* variant V279. Information-borrowing map shows the amount of borrowing from neighboring variants for *PLA2G7* variant V279 for different values of *c*, with darker color representing higher levels of contribution via the variant correlation matrix *R*.

In Fig 3 we show the relative 3D positions of the 13 rare variants in *PLA2G7*, focusing on variant V279 (highlighted in green). Each other variant is colored to indicate the magnitude of contribution into the local genetic similarity for V279 over different values of *c*, with those colored in lighter pink having lower contribution, and darker pink indicating higher contribution. In agreement with Table 1, we see that when *c* = 0.1, V279 does not significantly borrow information from any neighbors. When *c* = 0.3, it begins borrowing information from its closest neighboring variant, L283 (*r*_*L*283,*V*279_ ≈ 0.2). For *c* = 0.5, this amount increases (*r*_*L*283,*V*279_ ≈ 0.5), and we also see marginal contributions from M331 (*r*_*M*331,*V*279_ ≈ 0.1). As *c* increases beyond 0.5 (e.g., *c* = 0.7), V279 begins to borrow information from variants spaced further away on the protein. For larger values of *c* (e.g., *c* ≥ 2), the local test approaches a global test using similar weights for all variants along the protein. Similar patterns are observed when other variants are set as focal variants, as shown in S1 Fig.

#### Selection performance of risk variants

We first examine how each method behaves under the null hypothesis of no causal variants. S2 Fig shows the quantile-quantile plots of the p-values for SVT, SCAN, ADA, REBET, and POINT (applied using burden and linear local kernels and denoted by POINT-Burden and POINT-Linear, respectively) for both quantitative traits (top) and binary traits (bottom). The quantile-quantile plots compare the observed p-values with the expected p-values under the null. For all methods, the points fall near the 45 degree line, confirming the validity and appropriate implementation of each method.

We summarize the selection performance of each method with a p-value threshold of 0.05 under different causal scenarios for continuous traits in Table 2 (*n* = 2000) and S3 Table (*n* = 1000), which includes SVT, REBET, POINT-Burden and POINT-Linear. The results for binary traits are given in Table 3 (*n* = 2000) and S4 Table (*n* = 1000), which additionally includes SCAN and ADA. In the tables, the method with the best performance (based on the composite F-measure) are shown in bold and the second best are shown in *italic*.

Although more methods are considered in the binary-trait simulations, the relative performance among different methods are similar for both trait types and can be summarized into the following points. First, when only one variant is causal (i.e., Scenarios (D69), (F110), and (K191)), SVT as expected has the best performance, followed by POINT-Burden or POINT-Linear. Second, for a larger sized causal cluster (i.e., Scenario (G303, A326, M331)), REBET and SCAN have the best performance, followed by POINT-Burden. Third, for the rest of the scenarios, POINT-Burden has the best performance, followed by POINT-Linear (in most scenarios) or REBET (e.g., under Scenario (D69, R82)+(G303, A326, M331) with *n* = 2000). These scenarios include causal variants from a small cluster (i.e., Scenarios (D69, R82), (F110, S273) and (K191, D200)), from a subset of a true cluster (i.e., Scenario (A326, M331)), and from two separate clusters (i.e., Scenario (D69, R82)+(G303, A326, M331)). POINT-Burden has better or similar performance compared to POINT-Linear because the trait values are simulated assuming additive, equal-size effects; consequently, local burden collapsing is a more efficient kernel to capture the association effects in the simulation studies. The general patterns observed do not seem to vary under different causal allele frequencies changes or under different sample sizes.

We also examine the relative selection performance of the methods over different selection criteria using ROC curves. The results are shown in S3 and S4 Figs (for continuous traits of *n* = 2000 and 1000, respectively) and S5 and S6 Figs (for binary traits of *n* = 2000 and 1000, respectively). The ROC plots evaluate the true positive rate of variant selection (sensitivity) of the test against the false positive rate of variant selection (1-specificity) over all possible ranges of selection thresholds. Better selection methods have larger area under the ROC curve, with plots approaching upper the upper left corner, where more causal variants are selected with fewer null variants selected. Generally speaking, we observe a similar pattern of relative performance over the possible range of selection thresholds as what seen in Tables 2 and 3. The only exception is the performance of REBET under Scenario (D69, R82)+(G303, A326, M331), where REBET becomes the fourth best method based on ROC curve while is the second best method based on the F measure. Under this scenario, REBET has much higher true positive rates, slightly higher false discovery rates, and much higher false positive rates compared to other methods, which results in a less desirable ROC curve performance.

In summary, while different methods have the best performance under different scenarios, POINT consistently yields either the best or comparable performance to the best methods. Given the underlying effect mechanism is unknown in practice, POINT may serve as a robust strategy to prioritize important rare variants after global association signals are detected.

### Results of application to the ACCORD study

#### Association analysis of LDL vs. *PCSK9*

**Fig 4:**
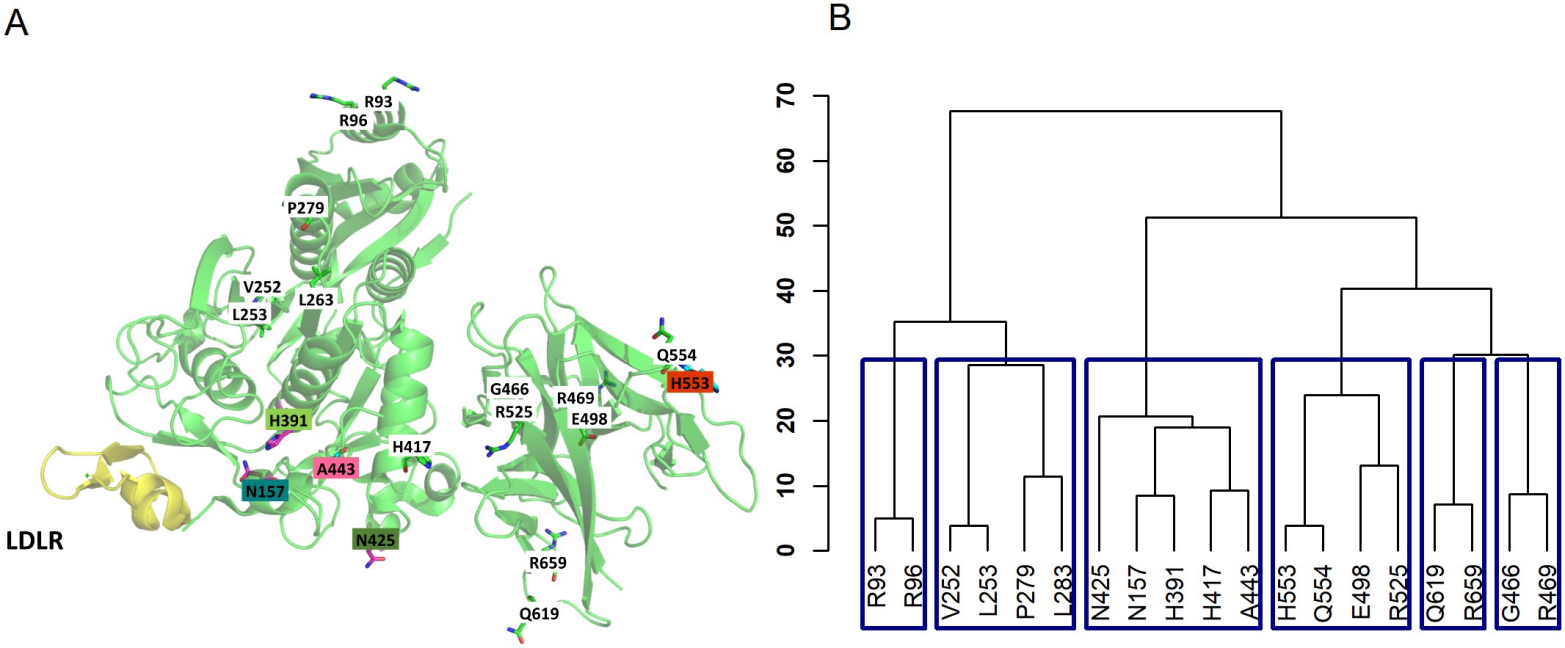
*PCSK9* rare variant positions. A: Rare variant locations on the protein tertiary structure of *PCSK9* binding with *LDLR* (shown in yellow). Promising variants (i.e., p-value<0.05) are shown in colored boxes, with variants found by both single variant test and POINT shown in red and variants found only by POINT shown in green and blue. B: Euclidean distance-based clustering of the variants.

Table 4 shows the analysis results for *PCSK9* using SVT, REBET and POINT. We select those variants with p-value less than 0.05 as promising variants. Using this criterion, there are two promising variants identified using both of SVT and POINT: A443 and H553. POINT further identifies three additional variants: N157, H391, and N425. REBET reports the union of subregions 1∼4 as the region most significantly associated with LDL, which includes 4 of the POINT-identified variants. Fig 4A shows the variant positions on the 3D protein structure, with the two variants found by SVT and POINT in pink and red, and those found only by POINT in green and blue. Fig 4B shows the distance-based clustering of *PCSK9* variants based on their positions in the 3D protein structure.

**Table 4:** *PCSK9* analysis results summary. Results of *PCSK9* analysis using the single variant test (SVT), REBET, and POINT (POINT-Burden). Promising variants are selected using the criterion of p-value< 0.05 and are shown in bold font.

Variant ID (AA Coord.)	SNP RSID	SVT p-value	REBET p-value	POINT-Burden		
				Best *c*	p-value	Variants borrowed information from*
R93	rs151193009	0.159	**0.001**	0	0.195	N/A
R96	rs185392267	0.397		0.5	0.391	R93
**N157**	rs143117125	0.213		0.4	**0.023**	H391, A443
V252	rs149139428	0.554		0.5	0.147	L253, L283
L253	rs72646508	0.130		0.5	0.135	V252
P279	rs72646509	0.318		0.4	0.408	L283
L283	rs72646510	0.637		0.5	0.262	V252, P279, H391
**H391**	rs146471967	0.091		0.3	**0.010**	N157, A443
H417	rs143275858	0.318		0.5	0.106	N157, H391, A443, R525
**N425**	rs28362261	0.051		0.5	**0.034**	N157, A443
**A443**	rs28362263	**0.043**		0.5	**0.048**	N157, H391, H417, N425
G466	rs72646517	0.988	Not Significant	0.5	0.379	R469, E498
R469	rs141502002	0.225		0.5	0.237	G466, E498
E498	rs145468572	0.317		0.5	0.153	G466, R469, R525
R525	rs140286279	0.347		0.4	0.374	H417, E498
**H553**	rs28362270	**0.016**		0	**0.024**	N/A
Q554	rs149311926	0.124		0	0.194	N/A
Q619	rs28362277	0.183		0	0.218	N/A
R659	rs147182054	0.201		0	0.359	N/A

*: Only those variants with variant correlation matrix entry *r*_*ℓm*_ ≥ 0.05 are listed.

S7 Fig shows the amount of information borrowed from neighboring variants for each variant at their chosen best value of *c*. We see that many of the promising variants identified by POINT cluster close together and choose to borrow information from one another. In particular, we see mutual borrowing of information between N157, H391, and A443, with A443 also borrowing from H417 and R525. The plots also show how information sharing between neighboring variants does not have to be symmetric. An example is H417, who chooses to borrow information from variants H391 and A443, though does not contribute to H391. The patterns of borrowing show how information sharing is variant-specific, allowing each variant to choose whether or not to borrow information based on how consistent the prior set by the local kernel is with the raw data. We further see that large association signal can occur without needing or choosing to borrow information from neighboring variants, as is the case with the selected variant H553, which chooses not to borrow from nearby variant Q554.

It has been shown that *PCSK9* impacts LDL levels by binding with *LDLR* (low-density lipoprotein receptor), prohibiting *LDLR* from binding LDL, and leading to increased LDL plasma levels [48]. As shown in Fig 4A, the variants newly identified by POINT were the closest variants in *PCSK9* to the protein-binding domain of *PCSK9* and *LDLR*. To better understand the biological implications of these identified variants, we further examine whether the relevant mutant sequences could have significant impact on the PCSK9-LDLR binding stability compared to the wildtype sequence using MDS with 3 replicates for each sequence. We measure the atomic mobility for the wildtype protein and for each of the PCSK9 mutant proteins via per-residue root mean square fluctuation (RMSF). We then quantify the stability changes for the PCSK9-LDLR interaction by calculating the RMSF difference between the mutant and the wildtype, and use the Wilcoxon rank-sum test to detect any significant differences.

**Fig 5:**
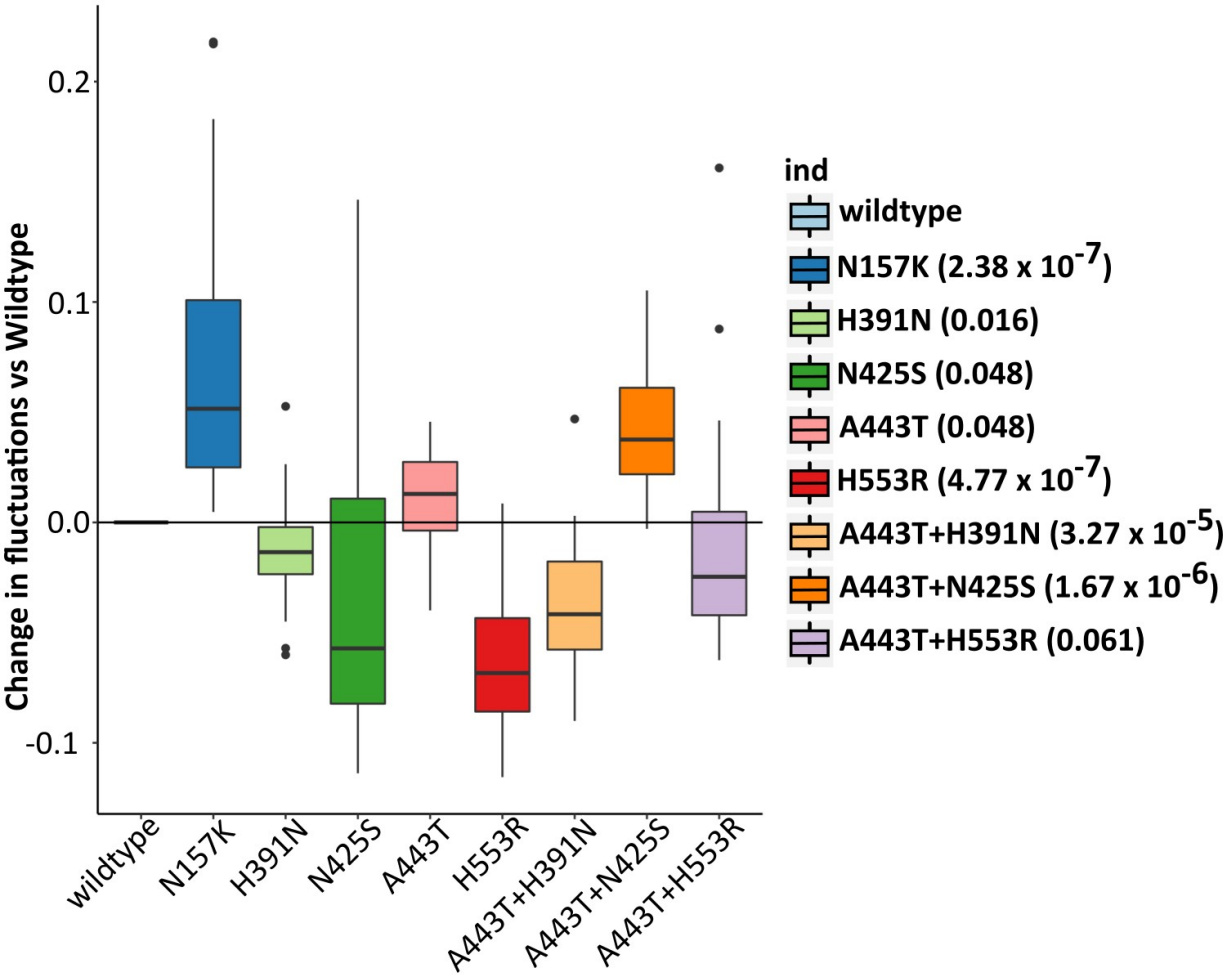
Assessment of *PCSK9-LDLR* binding stability for the mutant sequences from the five POINT-identified loci using molecular dynamic simulations. There are 8 mutant sequences observed in ACCORD samples, four of which have significant conformational fluctuation changes comparing to wildtype sequence: N157K, H553R, A443T+H391N, and A443T+N425S. P-values from Wilcoxon rank sum test of difference in RMSF are shown in parentheses.

Based on the five variants identified using POINT, there are 8 unique mutant sequences observed in the ACCORD samples as listed in Fig 5. Importantly, 4 out of the 8 mutant sequences have significant conformational changes in protein-protein interaction when compared to the wildtype: single mutations N157K (p-value 2.38 × 10^−7^) and H553R (p-value 4.77 × 10^−7^), and double mutations A443T combined with H391N (p-value 3.27 × 10^−5^) and A443T combined with N425S (p-value 1.67 × 10^−6^). The RMSF changes for the eight mutant sequences and corresponding p-values are shown in Fig 5. We note that a negative RMSF difference indicates that the amino acids involved in the protein-protein interaction have coordinates that fluctuate less than that of the wildtype (and hence the interaction is classically expected to be stronger). In contrast, a positive RMSF difference indicates that the amino acids move more than that of the wildtype (and hence the overall protein-protein interaction is expected to be weaker). Three of the significant sequences (i.e., N157, A443+H391, and A443+N425) are only identified by POINT. These results suggest a potential biological impact of these POINT-identified variants on the *PCSK9-LDLR* binding stability and hence an effect on the LDL level.

#### Association analysis of HDL vs. *ANGPTL4* and *CETP*

The results of the *ANGPTL4* analysis are summarized in S2 Appendix. SVT and POINT-Burden identify variant G223; POINT additionally identifies E190 and Q331; REBET reports Subregion 1 (the most significant) and Subregions 2+3 as significant; these regions include all POINT-identified variants. Among the genotyped variants in *ANGPTL4* with the ACCORD samples, the subdomain consisting of V308, G321, Q331 and R336 has been suggested to be a site for ligand binding (Biterova et al. [49]). We see that both SVT and POINT yield a p-value near 0.05 for R336; POINT additinoally selects Q331, and REBET appears to capture the signal in Subregions 2+3. The importance of G223 has been reported recently in Biterova et al. [49] and in phylogenetic analysis (Maxwell et al. [50]), suggesting the effect on folding and/or stability of *ANGPTL4* and strong relaxation from purifying selection. E190 has been reported to be associated with low plasma triglyceride levels and defines the plasma triglyceride level quantitative trait locus (TGQTL) in UniProt (Entry Q9BY76: Variant p.Glu190Gln).

The results of *CETP* analysis are summarized in S3 Appendix. SVT and POINT-Burden identify T61; POINT additionally identifies Q128; REBET identifies Subregion 1, which contains both POINT identified variants. Both T61 and Q128 have been reported to have deleterious and predicted damaging effects (e.g., Dergunov et al. [51].) Literature suggested that R299 and V340 participate in the lipid binding site with cholesteryl ester, triglyceride, and phospholipid (Qiu et al. [52]) but none of the methods identify association signals in the ACCORD dataset.

We note that the rare variants identified here are based on ACCORD samples, which consist of diabetic patients. The population is different from other studies (e.g., Romeo et al. [53]) that included more ethnically diverse populations. It would be an interesting next step to try to replicate our results in these cohorts. Nevertheless our results take the rare-variant mapping to a new level of details by identifying specific rare variants that may play a role.

## Discussion

In this work, we introduce an analytic framework, POINT, to identify promising variants that may be responsible for the association signals identified by global tests. POINT prioritizes rare variants by incorporating protein 3D structure to guide local collapsing analysis. With POINT, we introduce a mathematical formulation of tertiary protein structure using a structural kernel, develop a statistical framework to perform inference at a localized level guided by the protein structure, and describe how the structure-supervised analysis can be used to identify variants likely to have an effect on the trait of interest. The performance of POINT is robust and stable across different scenarios investigated in this study. POINT has similar or improved selection performance to identify risk rare variants compared to alternate methods, i.e., SVT, SCAN, ADA, and REBET. We have implemented the proposed analyses in R package POINT, available at impact.unc.edu/point.

POINT is adaptive, utilizing a data-driven scale *c* and the minimum *p* statistic to determine (1) the appropriate neighboring variants to borrow information from, and (2) the optimal amount of information to borrow from those neighboring variants. As shown in the information-borrowing maps (Fig 3, S1 and S7 Figs), while neighboring variants do tend to borrow from one another to gain strength, this borrowing only occurs when the data are supportive of the prior suggested by the protein structure and the borrowing does not have to be symmetric between a pair of variants.

Applying POINT to the ACCORD clinical trial, we are able to pinpoint three new rare variants that are not found by single variant testing, all near the protein-binding domain between *PCSK9* and *LDLR*. The results highlight the strength of our integrative method to find additional signal that cannot be found by other methods considered in the study. This finding might have clinical impact, given that *PCSK9* inhibitors are a new class of drugs and are being accepted as a promising treatment for reducing LDL levels [54, 55]. However, we note that the POINT signals are identified based on “association”, and hence the selected variants may or may not be “causative” mutations. Additional follow-up studies will have to be determined by the particulars of the result, the overall goals of the study, and available resources for additional follow-up.

POINT is constructed under the kernel machine framework with three main considerations that may affect performance: (1) choice of kernel, (2) choice of PDB entry, and (3) choice of grid of *c* values. For the first consideration, as noted in the literature, the local kernel test is valid even if a “wrong” kernel is chosen [9]. However, the power can be significantly affected by the choice of kernels because different kernel functions represent different underlying effect mechanisms (e.g., whether neighboring causal variants have similar or different effect patterns). Because such effect mechanisms are unknown a priori, choosing the “correct” or “optimal” kernel is still an important open problem in general kernel machine regression. One way to ensure the use of a “near optimal” kernel is to apply the composite kernel of Wu et al. [9], which can yield performance similar to the optimal kernel with substantial improvement over “wrong” kernels.

For the second consideration, we detail a few criteria for choosing an optimal protein structure entry from PDB, including good data quality and high coverage. In this work, we illustrate the POINT analysis under the scenario that the variants’ positions in the 3D protein structure can be obtained from a single PDB entry. However, in practice it is possible that no single entry has high coverage for the desired variant set. In this case, one can obtain the coordinate information by aligning multiple PDB entries with overlapping mapped residues using the PyMOL software (The PyMOL Molecular Graphics System, Version 1.8, Schrödinger, LLC.). When a variant in the set has no known coordinate information, instead of excluding it from the analysis as we did here, one may choose to include the variant by setting its Euclidean distance to all other variants to be infinite, essentially using a single variant test for this variant.

In our analyses, we handle the third consideration by adaptively choosing a scale *c* over a grid ranging from *c* = 0 to *c* = 0.5. We show, using tables and variant borrowing maps, how the maximum *c* value affects how far the focal variant willing to borrow information from. We choose *c* = 0.5 as our maximum grid value to ensure borrowing only from neighbors who may be considered to cluster close together on the protein tertiary surface, as the literature suggests common effects from closely clustered variants. As this is a multiplier of the standard deviation of distance between variants, this choice should also be applicable to different protein structures. Choosing a larger maximum *c* may be considered, but with caution so as not to increase false signal which may arise from borrowing outside of the cluster.

**Fig 6:**
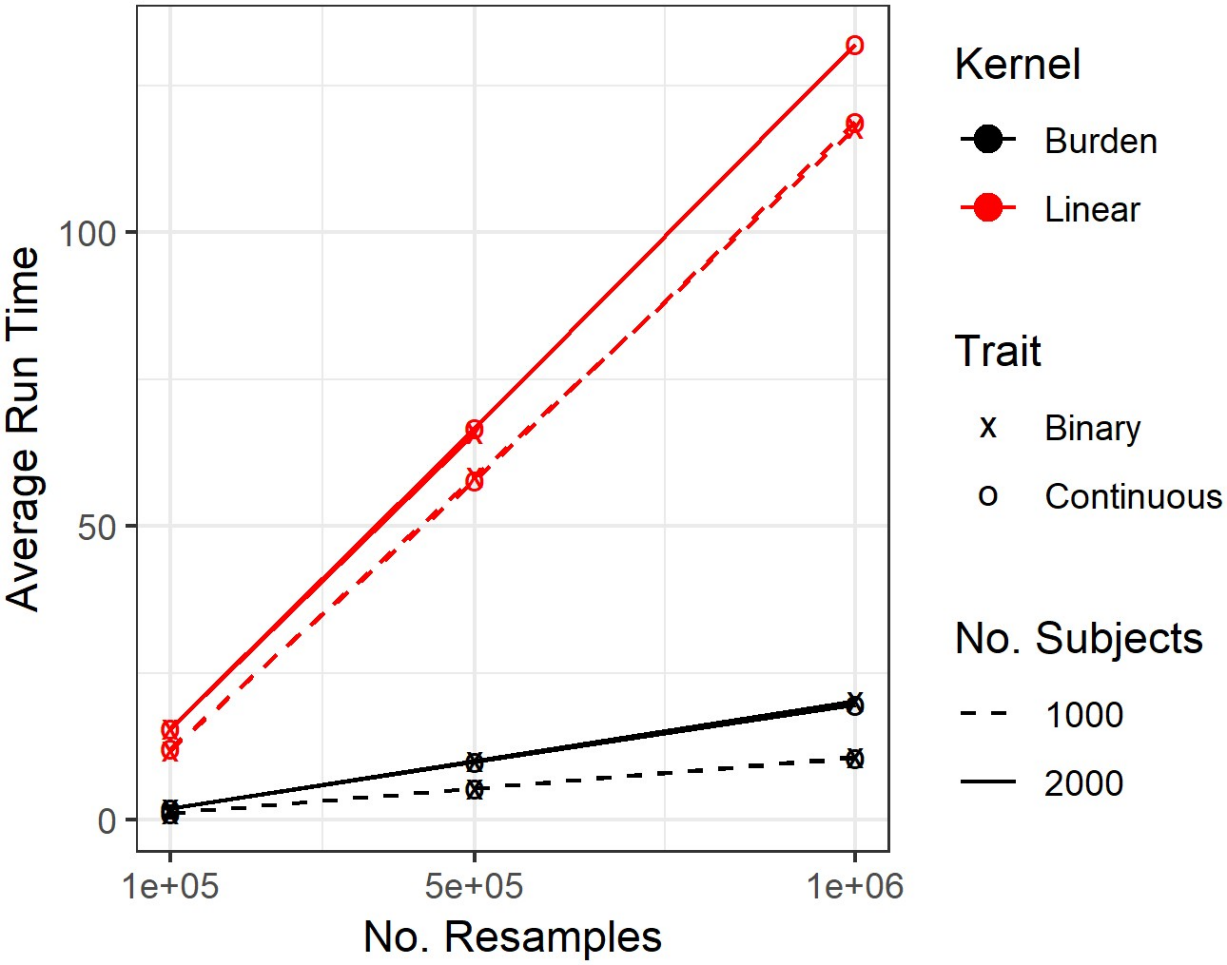
POINT computational time with large number of resamples, i.e., 1*e*5, 5*e*5 and 1*e*6. Average run time (in minutes) based on 10 replications for a single variant POINT test with 13 associated variants.

Finally, we comment on the computational cost of POINT. POINT uses a resampling approach to compute the p-value of the minP statistic that corresponds to the optimal *c*. In the numerical analysis here, we consider the number of resamples as 1000. In practice, a larger number of resamples may be needed in order to compute the p-values at desired precisions. The computational cost of POINT will increase when the number of resamples increases. In Fig 6, we report the computational time POINT required with different number of resamples. The computations are carried out on one core of the Dell R620 dual-Xeon (E5-2670, 2.60GHz) compute nodes with 128GB of RAM, and averaged over 10 replications per scenario. We found that the run time increases roughly linearly with the number of resamples for both continuous and binary outcomes and for both kernels. The computational time is roughly the same for continuous or binary outcomes; but the linear kernel requires substantially longer time (i.e.,∼10× longer than the burden kernel. In real practice, when POINT has to be applied on a large number of variants and a high precision of p-values are required, one can adopt a two-stage procedure to improve the computational efficiency (besides using parallel computing on different variants), i.e., first to conduct POINT with a smaller number of resamples, e.g., *B* = 1000, and then use the desired, higher number of resamples on those variants with Stage-1 p-values ≤ 1/*B*.

## Supporting information

S1 Fig Information-borrowing map for each variant in *PLA2G7*. Information-borrowing map shows the amount of borrowing from neighboring variants for each of the *PLA2G7* variants for different values of *c*, with darker color representing higher levels of contribution via the variant correlation matrix *R*.
(PDF)

S2 Fig Quantile-quantile plots of p-values for *PLA2G7* simulation study under the null hypothesis of no causal variants using different methods. SVT: single variant test; POINT-Burden: POINT test using local burden kernel; POINT-Linear: POINT test using local linear kernel; SCAN: scan statistic method (from the p-values of the best window); ADA of Lin (2016); REBET of Zhu et al. (2016). The top panel is for continuous traits and the bottom panel is for binary traits.
(TIFF)

S3 Fig Empirical receiver operating characteristic (ROC) curves for continuous outcomes with *n* = 2000 subjects under different scenarios of causal variants. The simulation scenarios are listed in Table 2. The Y-axis is the true positive rate (i.e., sensitivity) and the X-axis is the false positive rate (i.e., 1-specificity). Red dotted line: single variant test (SVT); blue dashed line: POINT test using local burden kernel; green dash-dot line: POINT test using local linear kernel; yellow solid line: REBET.
(TIFF)

S4 Fig Empirical receiver operating characteristic (ROC) curves for continuous outcomes with *n* = 1000 subjects under different scenarios of causal variants. The simulation scenarios are listed in Table 2. The Y-axis is the true positive rate (i.e., sensitivity) and the X-axis is the false positive rate (i.e., 1-specificity). Red dotted line: single variant test (SVT); blue dashed line: POINT test using local burden kernel; green dash-dot line: POINT test using local linear kernel; yellow solid line: REBET.
(TIFF)

S5 Fig Empirical receiver operating characteristic (ROC) curves for binary outcomes with *n* = 2000 subjects under different scenarios of causal variants. The simulation scenarios are listed in Table 3. The Y-axis is the true positive rate (i.e., sensitivity) and the X-axis is the false positive rate (i.e., 1-specificity). Red dotted line: single variant test; blue dashed line: POINT test using local burden kernel (SVT); green dash-dot line: POINT test using local linear kernel; purple short-dash line: scan statistic method (SCAN); red dashed line: ADA; yellow solid line: REBET.
(TIFF)

S6 Fig Empirical receiver operating characteristic (ROC) curves for binary outcomes with *n* = 1000 subjects under different scenarios of causal variants. The simulation scenarios are listed in Table 3. The Y-axis is the true positive rate (i.e., sensitivity) and the X-axis is the false positive rate (i.e., 1-specificity). Red dotted line: single variant test; blue dashed line: POINT test using local burden kernel (SVT); green dash-dot line: POINT test using local linear kernel; purple short-dash line: scan statistic method (SCAN); red dashed line: ADA; yellow solid line: REBET.
(TIFF)

S7 Fig *PCSK9* information-borrowing map. Information-borrowing map shows the amount of borrowing from neighboring variants for the chosen best *c* value for the local kernel test of association between the rare variants in *PCSK9* and LDL.
(TIFF)

S1 Table *PLA2G7* rare variant summary information. Minor allele frequency and protein coordinate information for the rare variants in *PLA2G7*. The 3D coordinates are obtained from PDB entry 3F96.
(PDF)

S2 Table *PCSK9* rare variant summary information. Minor allele frequency and protein coordinate information for the rare variants in *PCSK9*. The 3D coordinates are obtained from PDB entry 4K8R.
(PDF)

S3 Table Selection performance of continuous-trait simulations with *n* = 1000 subjects. Selection performance for single variant test (SVT), REBET, POINT test using local burden kernel (POINT-Burden), and POINT test using local linear kernel (POINT-Linear). The best performed methods (based on the composite F-measure) are shown in bold.
(PDF)

S4 Table Selection performance of binary-trait simulations with *n* = 1000 subjects. Selection performance for single variant test (SVT), scan statistic (SCAN), ADA, REBET, POINT test using local burden kernel (POINT-Burden), and POINT test using local linear kernel (POINT-Linear). The best performed methods (based on the composite F-measure) are shown in bold.
(PDF)

S1 Appendix Resampling approach to obtain the p-value of the localized test for variant *m*. (PDF)

S2 Appendix *ANGPTL4* rare variant summary information, locations on the protein tertiary structure, and results of association tests with high-density lipoproteins (HDL). (PDF)

S3 Appendix *CETP* rare variant summary information, locations on the protein tertiary structure, and results of association tests with high-density lipoproteins (HDL). (PDF)
