# Supplementary material for: Identifying individual risk rare variants using protein structure guided local tests (POINT)
Source: PLoS Comput Biol. 2019 Feb 19;15(2):e1006722. doi: 10.1371/journal.pcbi.1006722 (PMC6396946; doi:10.1371/journal.pcbi.1006722)
Supplement: S1 Fig — Information-borrowing map shows the amount of borrowing from neighboring variants for each of the PLA2G7 variants for different values of c, with darker color representing higher levels of contribution via the variant correlation matrix R. (PDF) [file pcbi.1006722.s001.pdf]

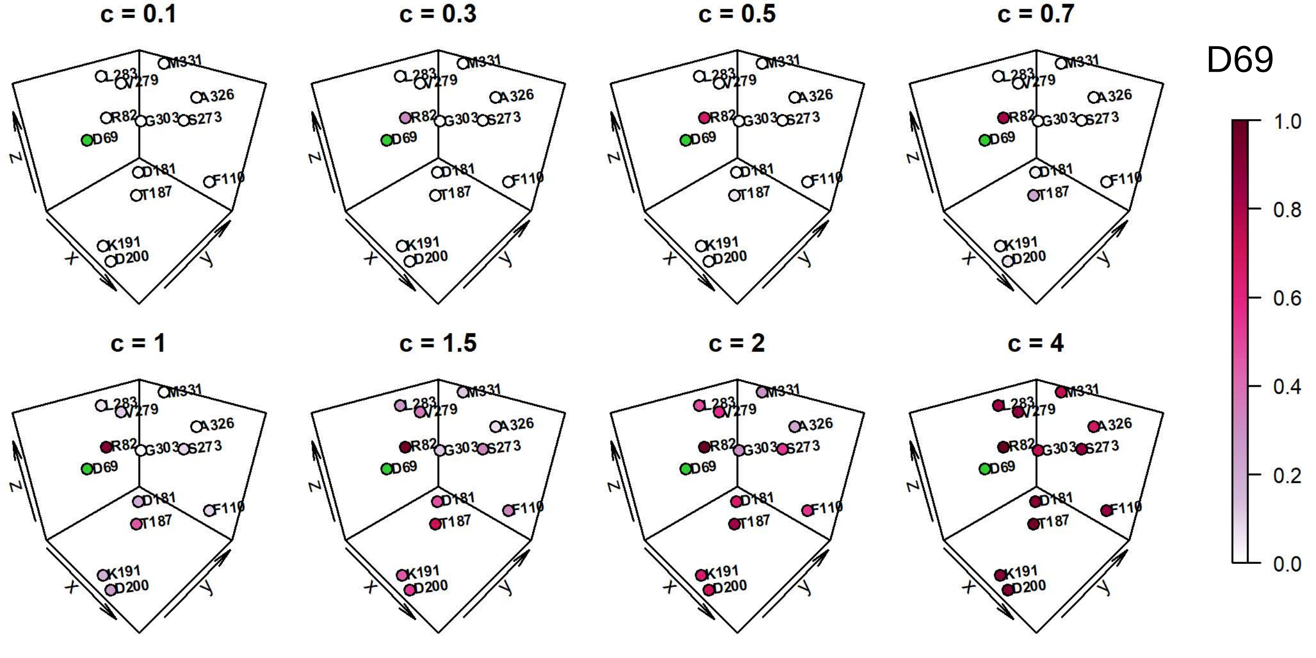

c = 0.1
c = 0.3
c = 0.5
c = 0.7
c = 1
c = 1.5
c = 2
c = 4
D69
L283
V279
M331
A326
R82
G303
S273
D181
F110
T187
K191
D200
x
y
z
1.0
0.8
0.6
0.4
0.2
0.0

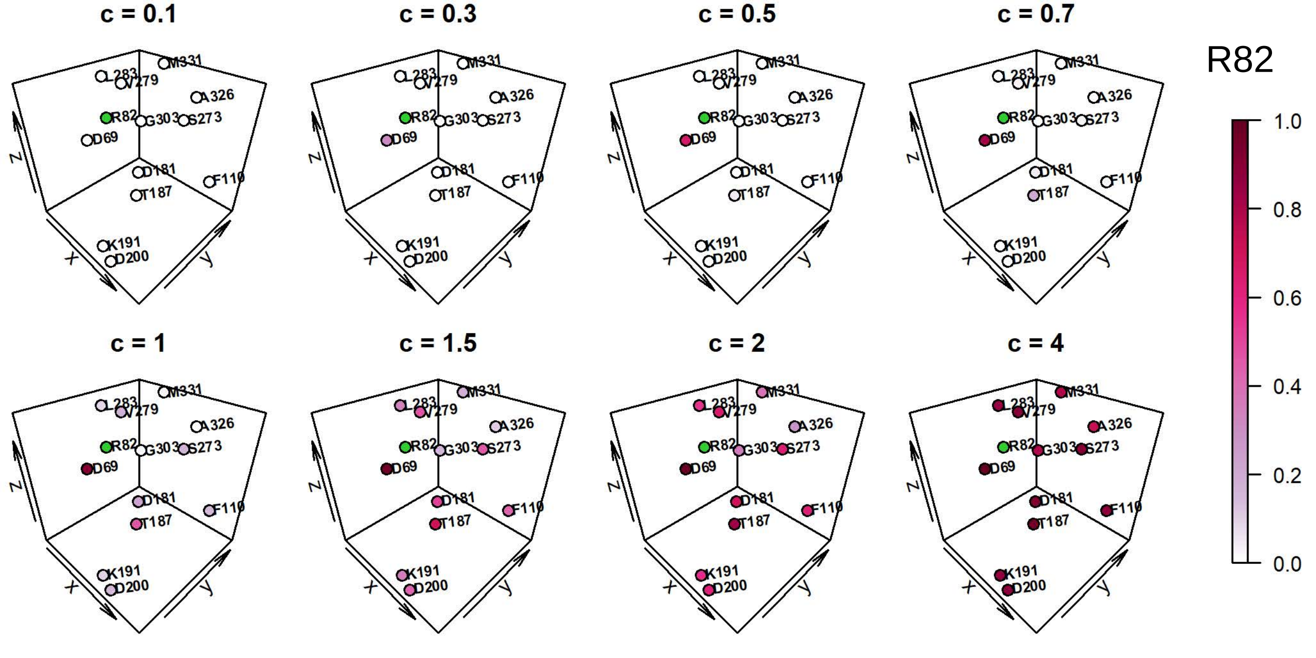

c = 0.1
c = 0.3
c = 0.5
c = 0.7
c = 1
c = 1.5
c = 2
c = 4
R82
L283
V279
M331
A326
G303
S273
D69
D181
F110
T187
K191
D200
x
y
z
1.0
0.8
0.6
0.4
0.2
0.0

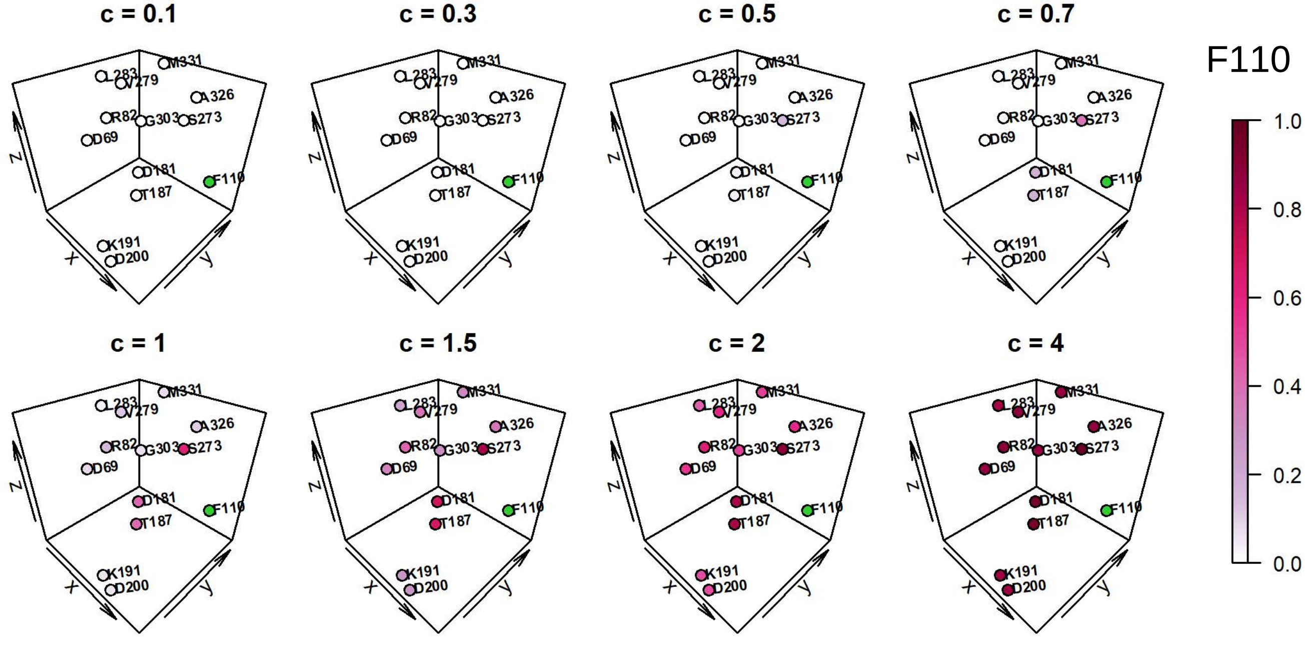

c = 0.1
c = 0.3
c = 0.5
c = 0.7
c = 1
c = 1.5
c = 2
c = 4
M331
L283
V279
A326
R82
G303
S273
D69
D181
F110
T187
K191
D200
x
y
z
F110
1.0
0.8
0.6
0.4
0.2
0.0

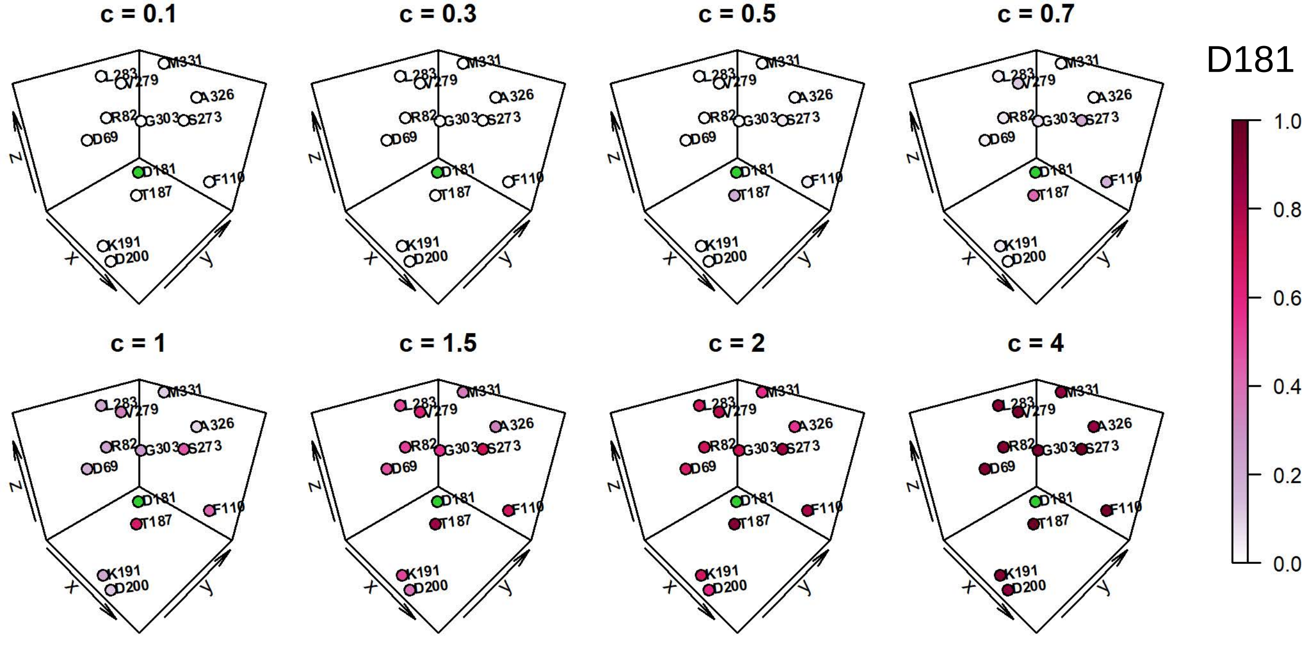
c = 0.1
c = 0.3
c = 0.5
c = 0.7
c = 1
c = 1.5
c = 2
c = 4
D181
M331
L283
V279
A326
R82
G303
S273
D69
D181
F110
T187
K191
D200
x
y
z
1.0
0.8
0.6
0.4
0.2
0.0

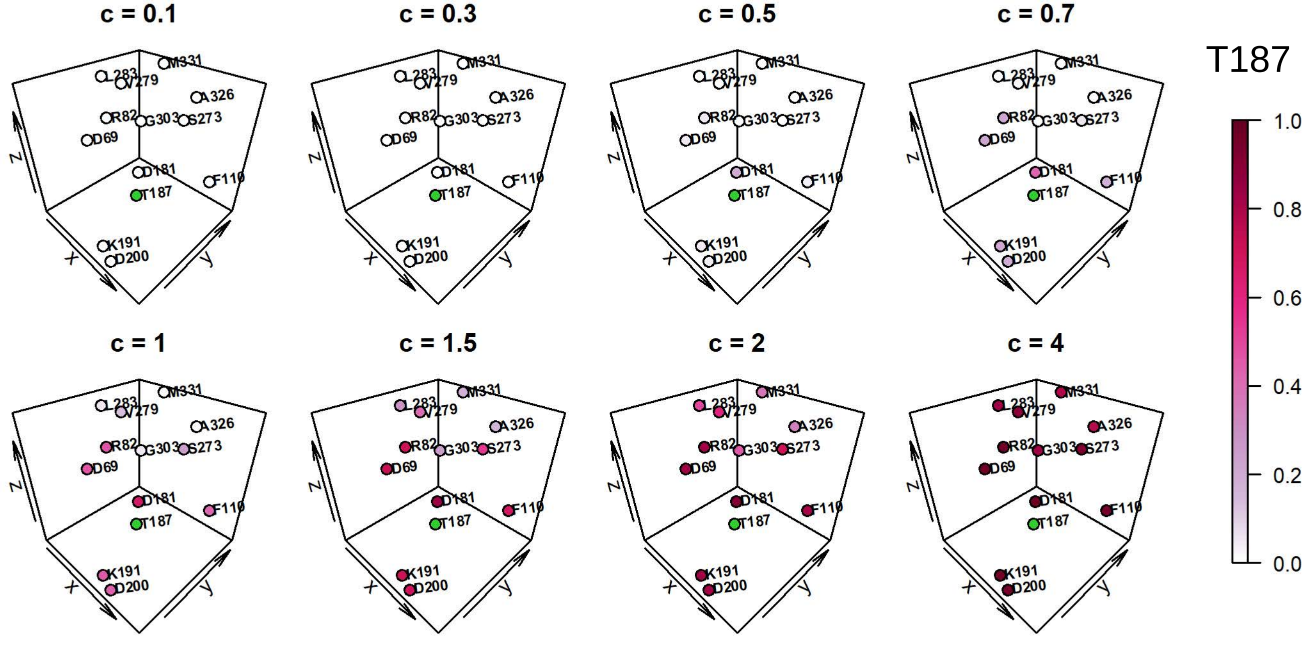
c = 0.1
c = 0.3
c = 0.5
c = 0.7
c = 1
c = 1.5
c = 2
c = 4
M331
L283
V279
A326
R82
G303
S273
D69
D181
F110
T187
K191
D200
x
y
z
T187
1.0
0.8
0.6
0.4
0.2
0.0

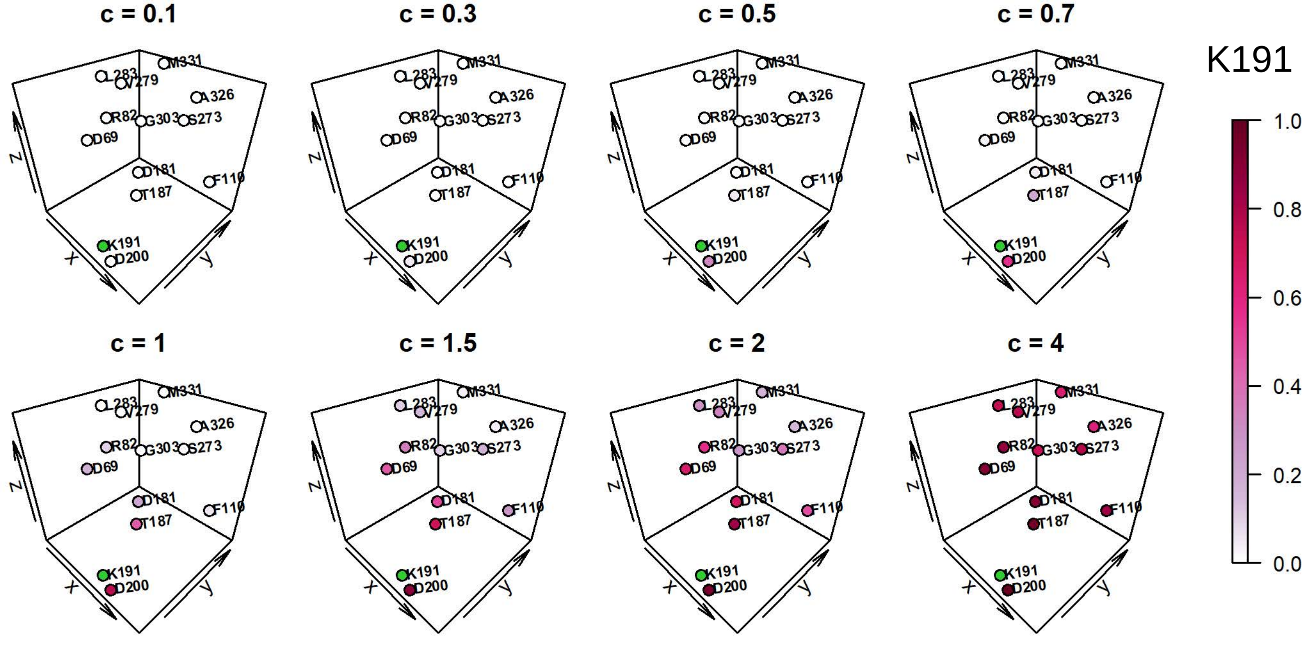
c = 0.1
c = 0.3
c = 0.5
c = 0.7
c = 1
c = 1.5
c = 2
c = 4
K191
M331
L283
V279
A326
R82
G303
S273
D69
D181
F110
T187
D200
x
y
z
1.0
0.8
0.6
0.4
0.2
0.0

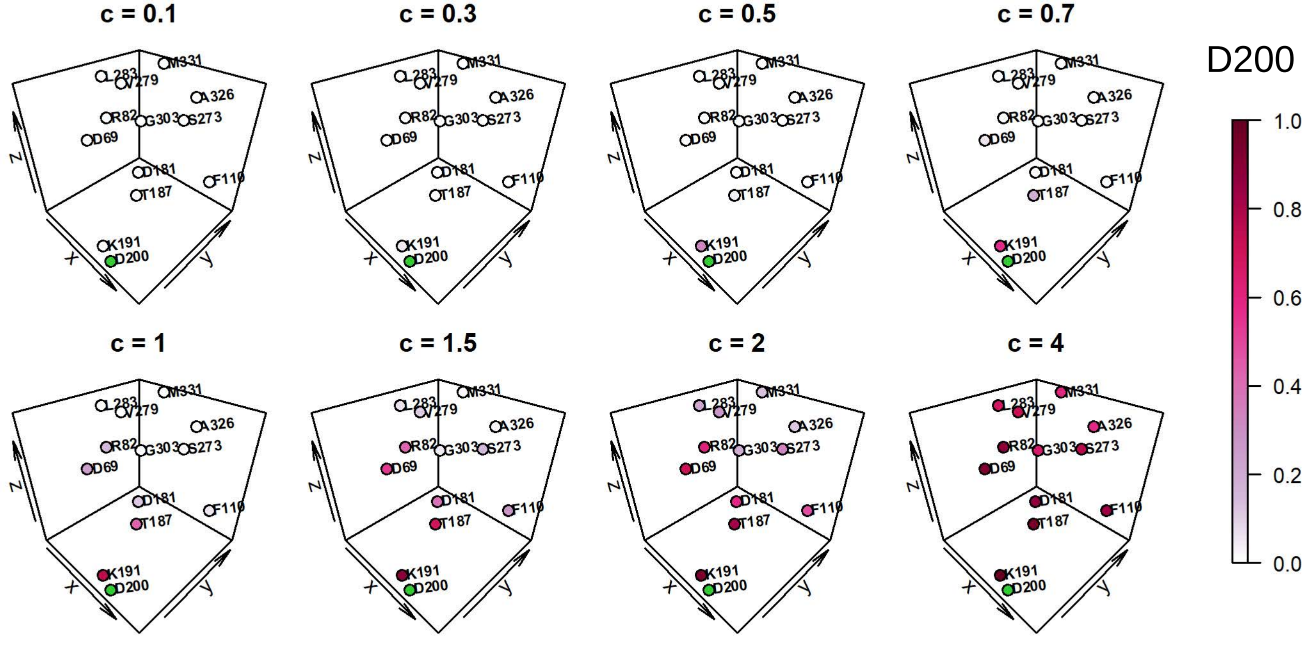
c = 0.1
c = 0.3
c = 0.5
c = 0.7
c = 1
c = 1.5
c = 2
c = 4
D200
L283
V279
M331
A326
R82
G303
S273
D69
D181
F110
T187
K191
x
y
z
1.0
0.8
0.6
0.4
0.2
0.0

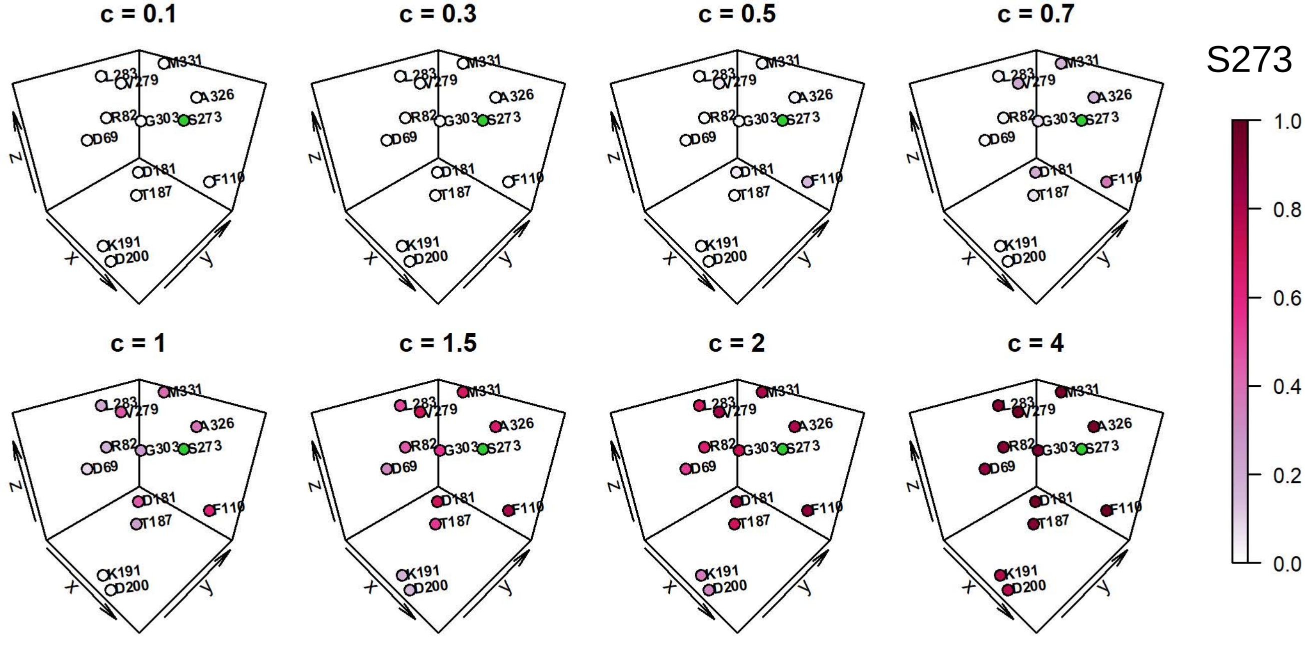
c = 0.1
c = 0.3
c = 0.5
c = 0.7
c = 1
c = 1.5
c = 2
c = 4
S273
M331
L283
V279
A326
R82
G303
S273
D69
D181
F110
T187
K191
D200
x
y
z
1.0
0.8
0.6
0.4
0.2
0.0

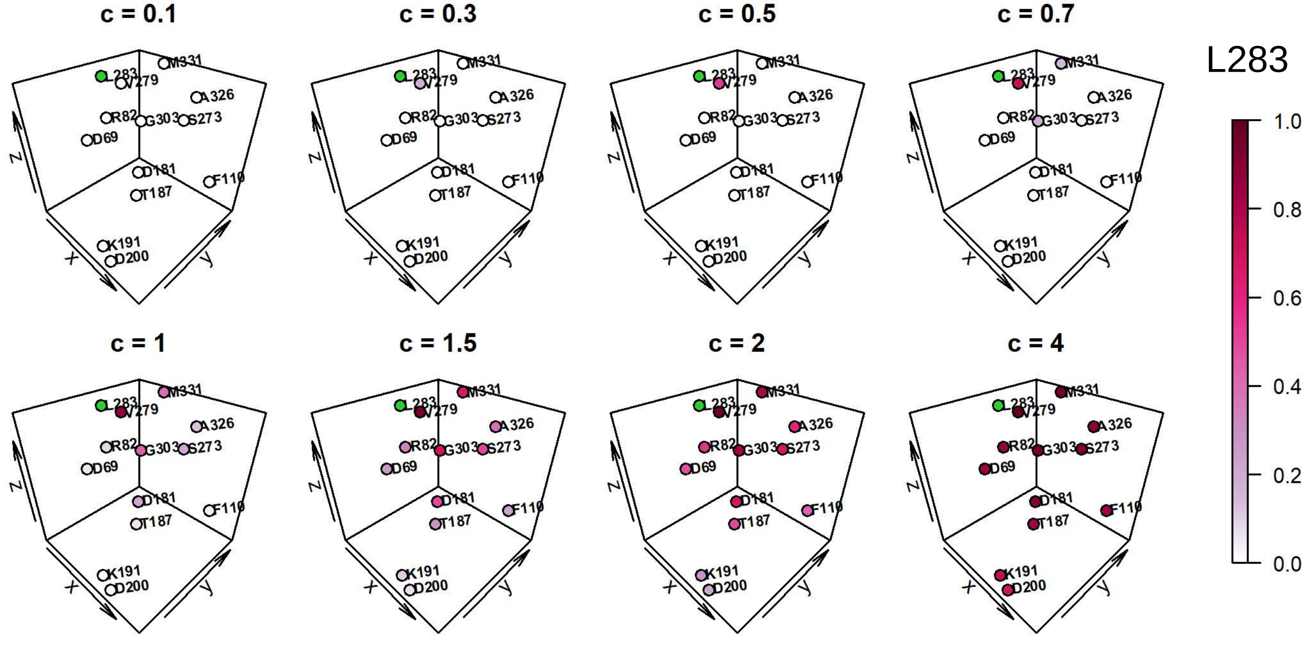

c = 0.1
c = 0.3
c = 0.5
c = 0.7
c = 1
c = 1.5
c = 2
c = 4
L283
V279
M331
A326
R82
G303
S273
D69
D181
F110
T187
K191
D200
x
y
z
1.0
0.8
0.6
0.4
0.2
0.0

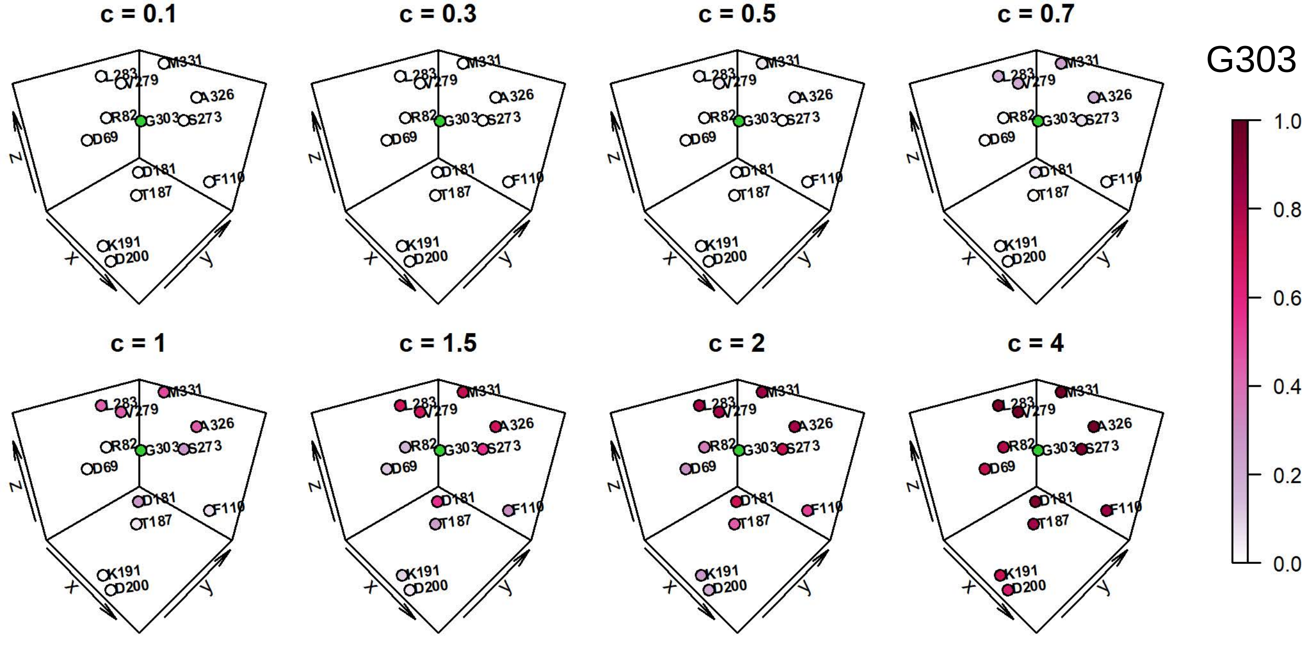

c = 0.1
c = 0.3
c = 0.5
c = 0.7
c = 1
c = 1.5
c = 2
c = 4
G303
L283
V279
M331
A326
R82
S273
D69
D181
F110
T187
K191
D200
x
y
z
1.0
0.8
0.6
0.4
0.2
0.0

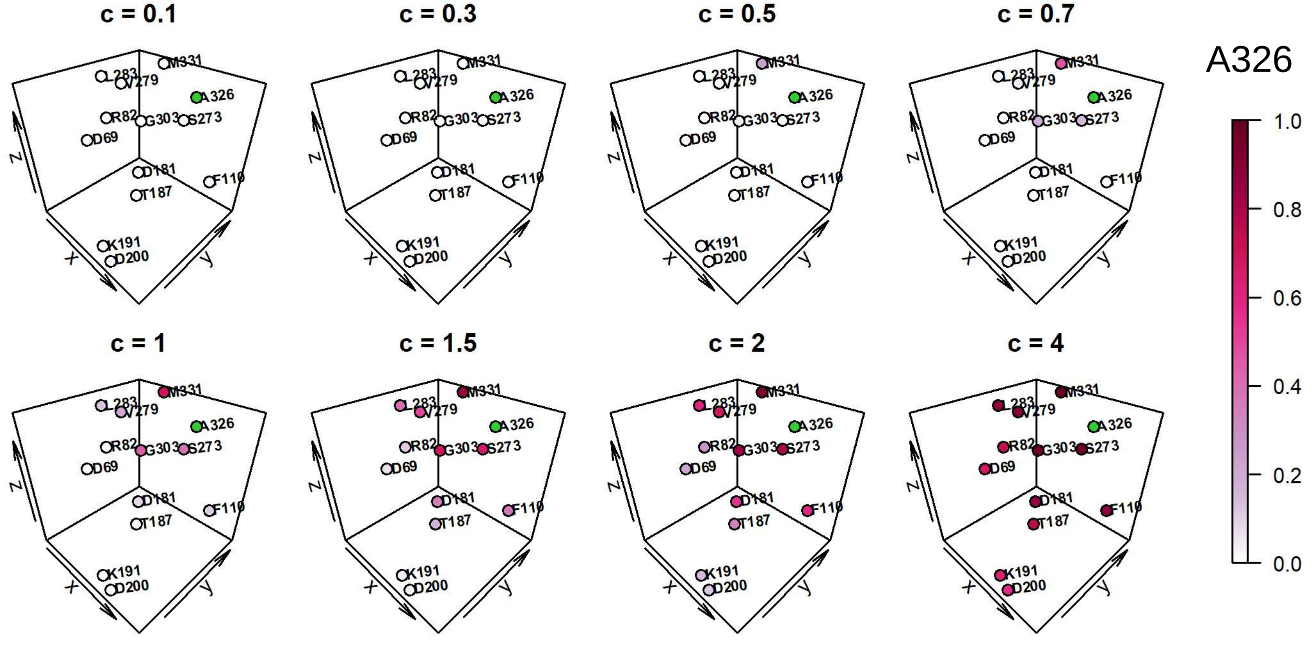

c = 0.1
c = 0.3
c = 0.5
c = 0.7
c = 1
c = 1.5
c = 2
c = 4
A326
M331
L283
V279
R82
G303
S273
D69
D181
F110
T187
K191
D200
x
y
z
1.0
0.8
0.6
0.4
0.2
0.0

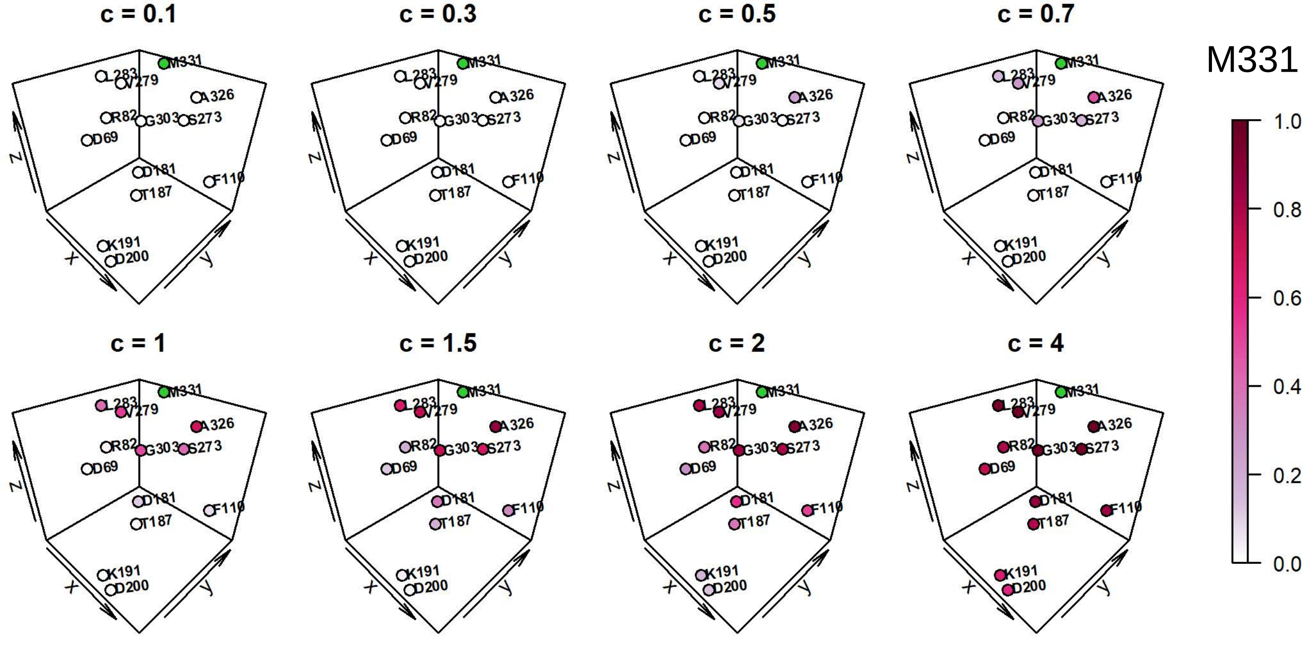
c = 0.1
c = 0.3
c = 0.5
c = 0.7
c = 1
c = 1.5
c = 2
c = 4
M331
L283
V279
A326
R82
G303
S273
D69
D181
F110
T187
K191
D200
x
y
z
1.0
0.8
0.6
0.4
0.2
0.0
