# Supplementary figures and images for: Identifying individual risk rare variants using protein structure guided local tests (POINT)
Source: PLoS Comput Biol. 2019 Feb 19;15(2):e1006722. doi: 10.1371/journal.pcbi.1006722 (PMC6396946; doi:10.1371/journal.pcbi.1006722)

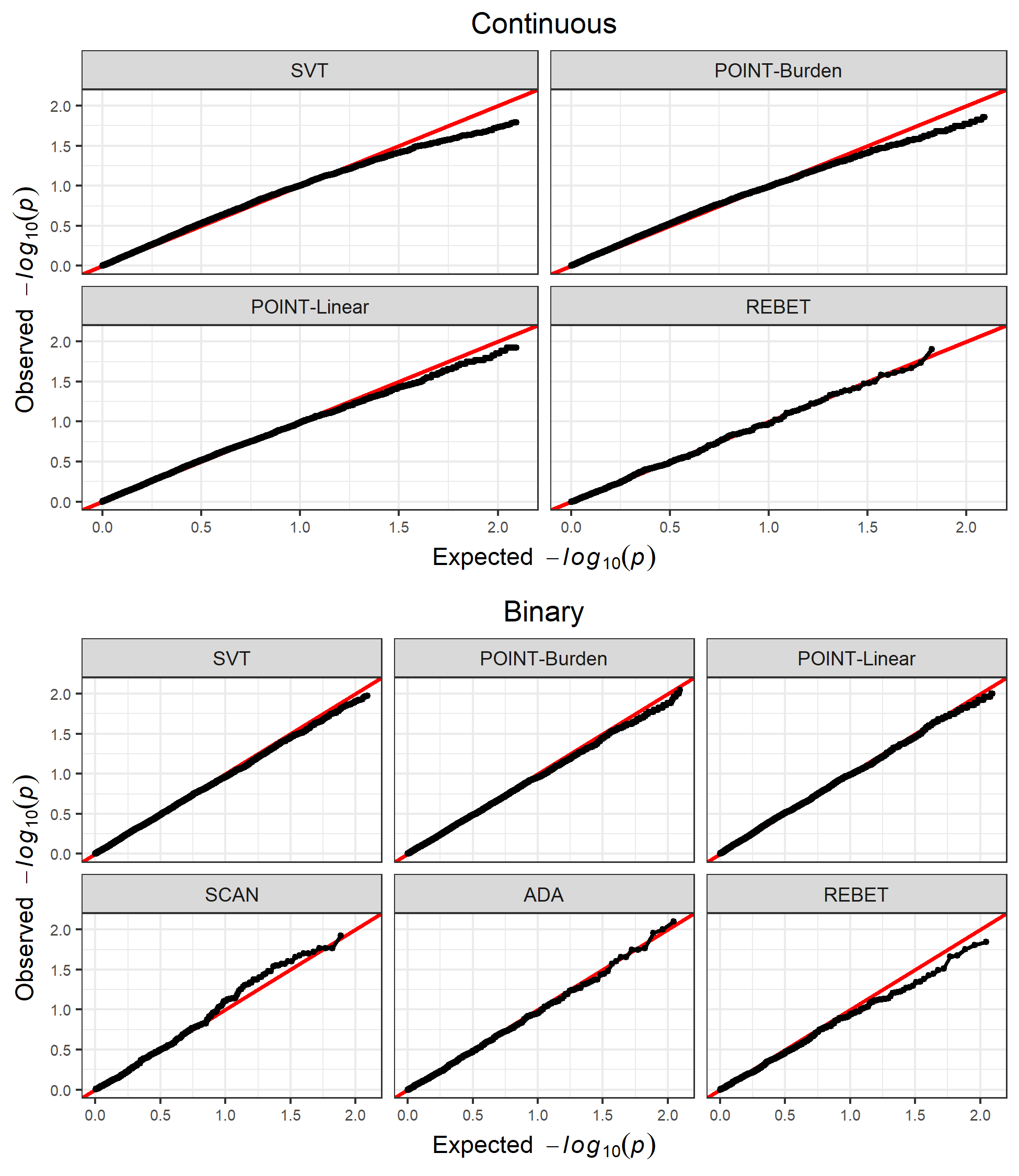

Supplement: S2 Fig — SVT: single variant test; POINT-Burden: POINT test using local burden kernel; POINT-Linear: POINT test using local linear kernel; SCAN: scan statistic method (from the p-values of the best window); ADA of Lin (2016); REBET of Zhu et al. (2016). The top panel is for continuous traits and the bottom panel is for binary traits. (TIFF) [file pcbi.1006722.s002.tiff]

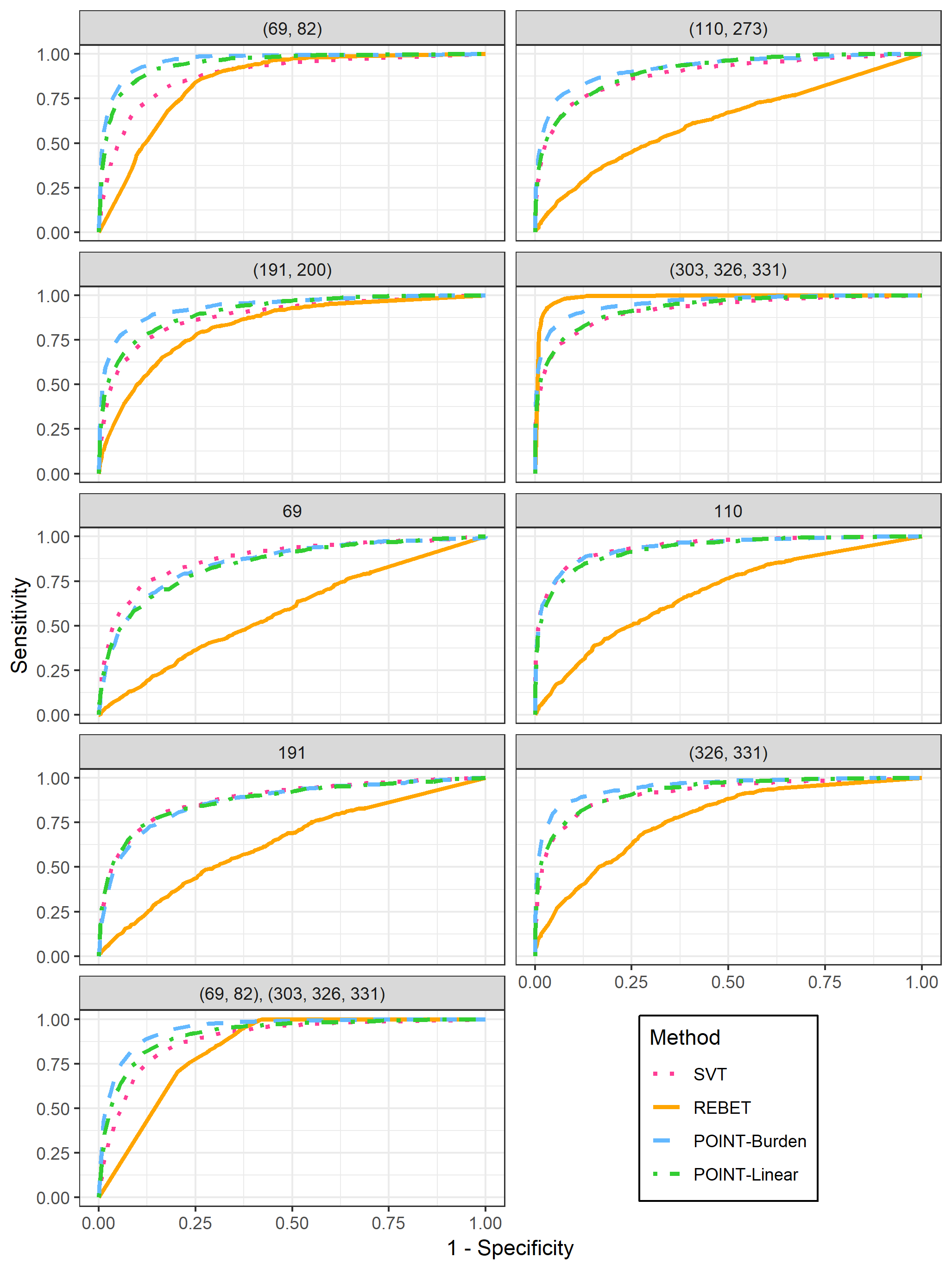

Supplement: S3 Fig — The simulation scenarios are listed in Table 2. The Y-axis is the true positive rate (i.e., sensitivity) and the X-axis is the false positive rate (i.e., 1-specificity). Red dotted line: single variant test (SVT); blue dashed line: POINT test using local burden kernel; green dash-dot line: POINT test using local linear kernel; yellow solid line: REBET. (TIFF) [file pcbi.1006722.s003.tiff]

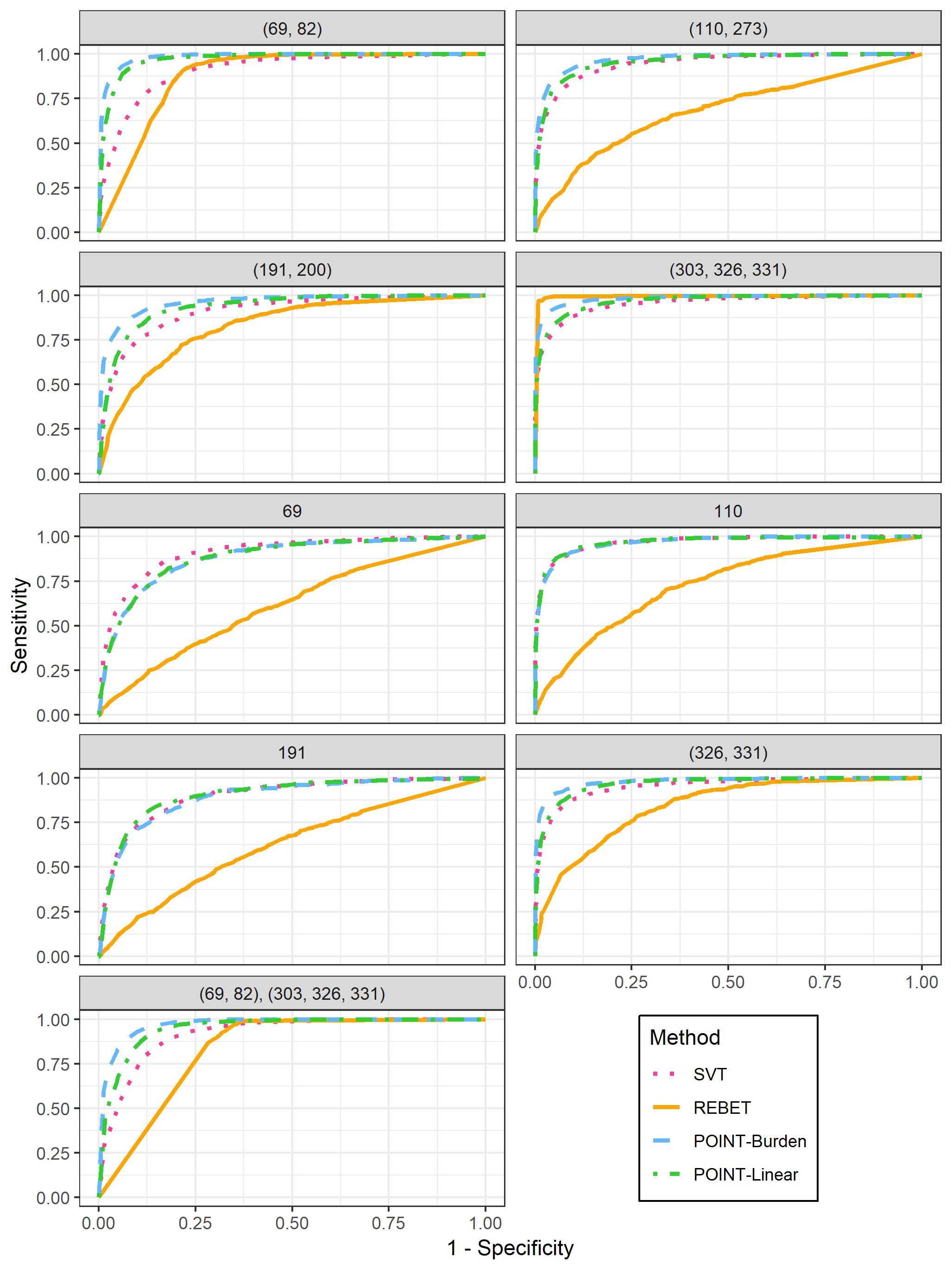

Supplement: S4 Fig — The simulation scenarios are listed in Table 2. The Y-axis is the true positive rate (i.e., sensitivity) and the X-axis is the false positive rate (i.e., 1-specificity). Red dotted line: single variant test (SVT); blue dashed line: POINT test using local burden kernel; green dash-dot line: POINT test using local linear kernel; yellow solid line: REBET. (TIFF) [file pcbi.1006722.s004.tiff]

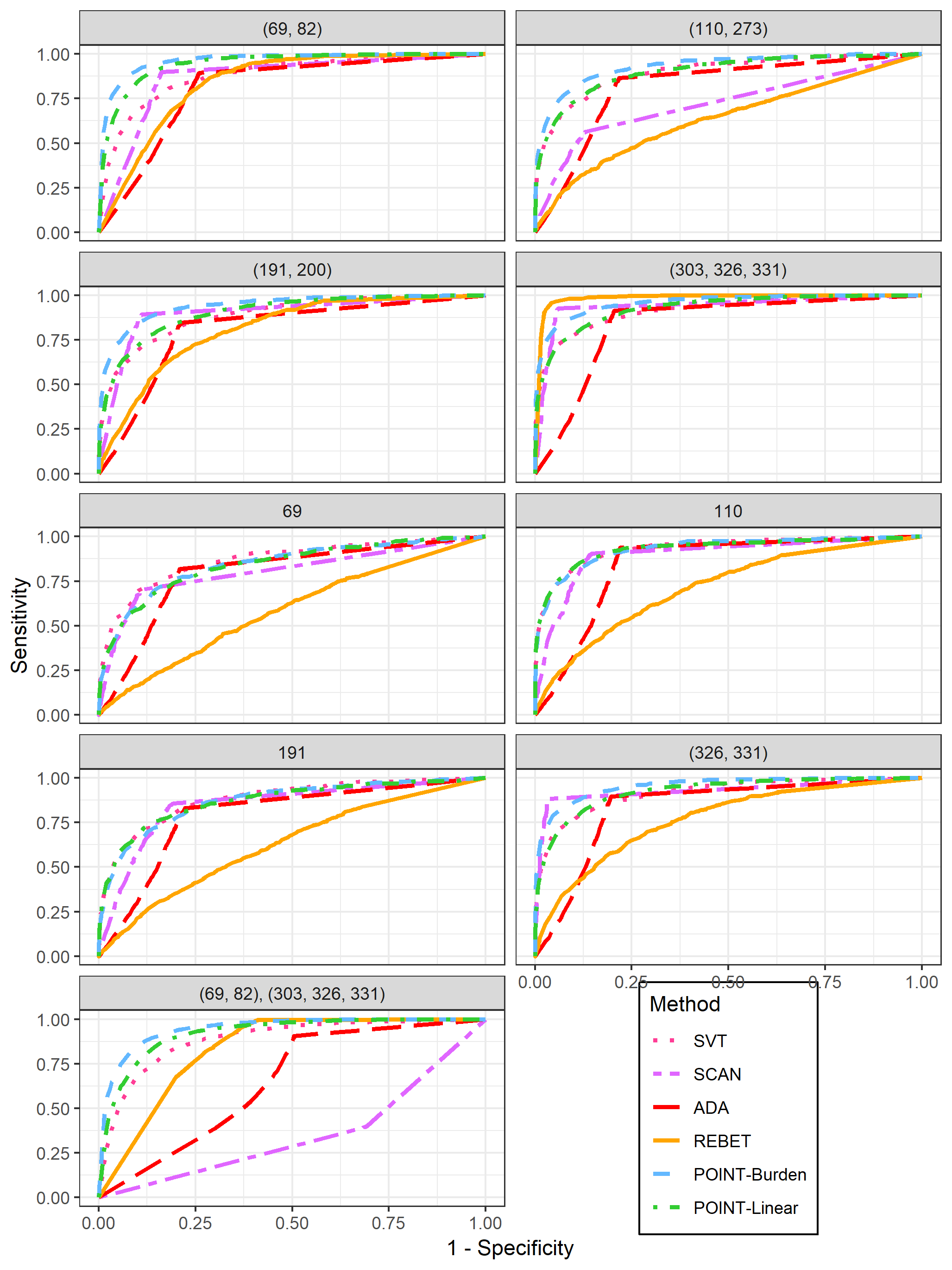

Supplement: S5 Fig — The simulation scenarios are listed in Table 3. The Y-axis is the true positive rate (i.e., sensitivity) and the X-axis is the false positive rate (i.e., 1-specificity). Red dotted line: single variant test; blue dashed line: POINT test using local burden kernel (SVT); green dash-dot line: POINT test using local linear kernel; purple short-dash line: scan statistic method (SCAN); red dashed line: ADA; yellow solid line: REBET. (TIFF) [file pcbi.1006722.s005.tiff]

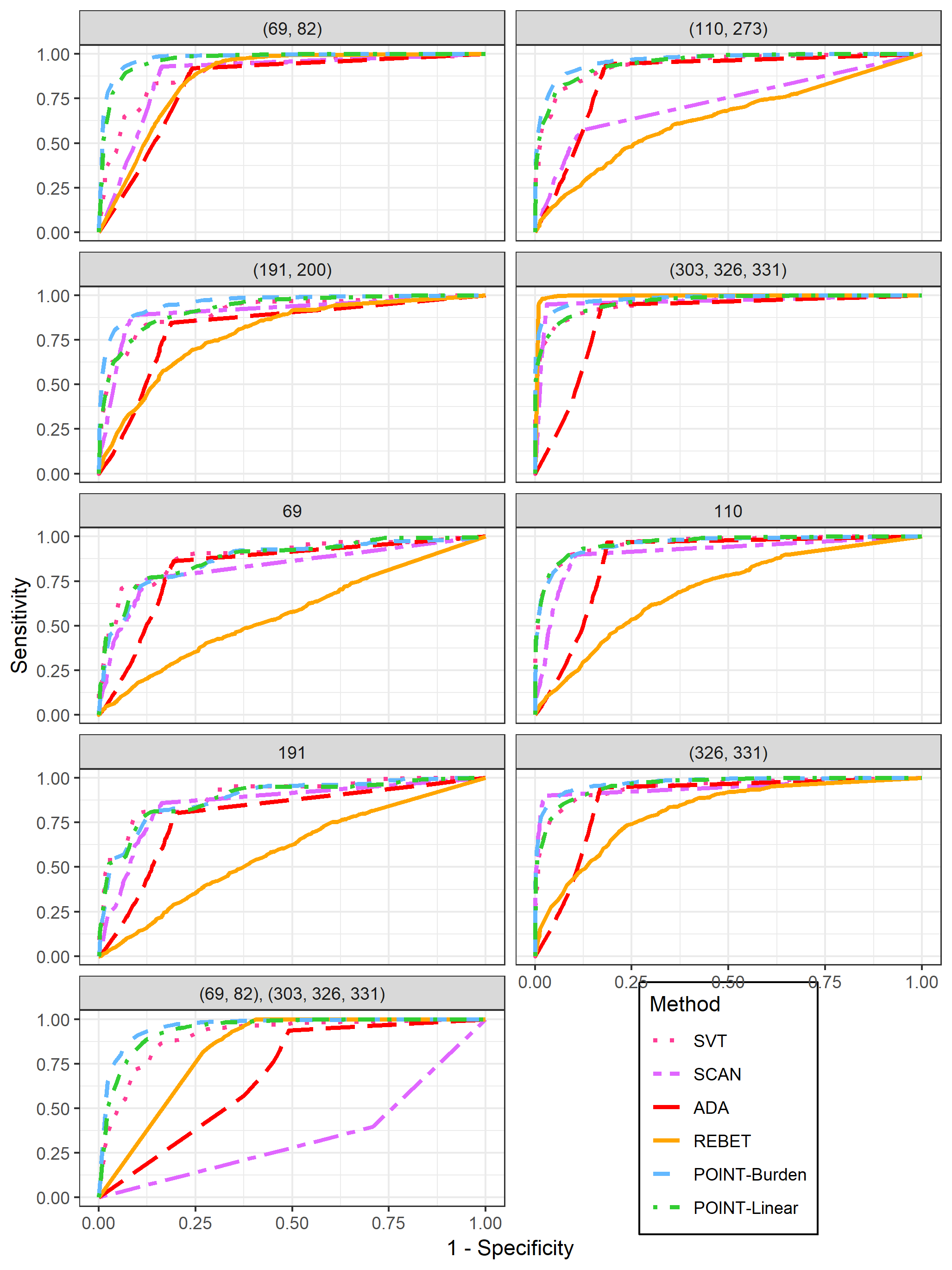

Supplement: S6 Fig — The simulation scenarios are listed in Table 3. The Y-axis is the true positive rate (i.e., sensitivity) and the X-axis is the false positive rate (i.e., 1-specificity). Red dotted line: single variant test; blue dashed line: POINT test using local burden kernel (SVT); green dash-dot line: POINT test using local linear kernel; purple short-dash line: scan statistic method (SCAN); red dashed line: ADA; yellow solid line: REBET. (TIFF) [file pcbi.1006722.s006.tiff]

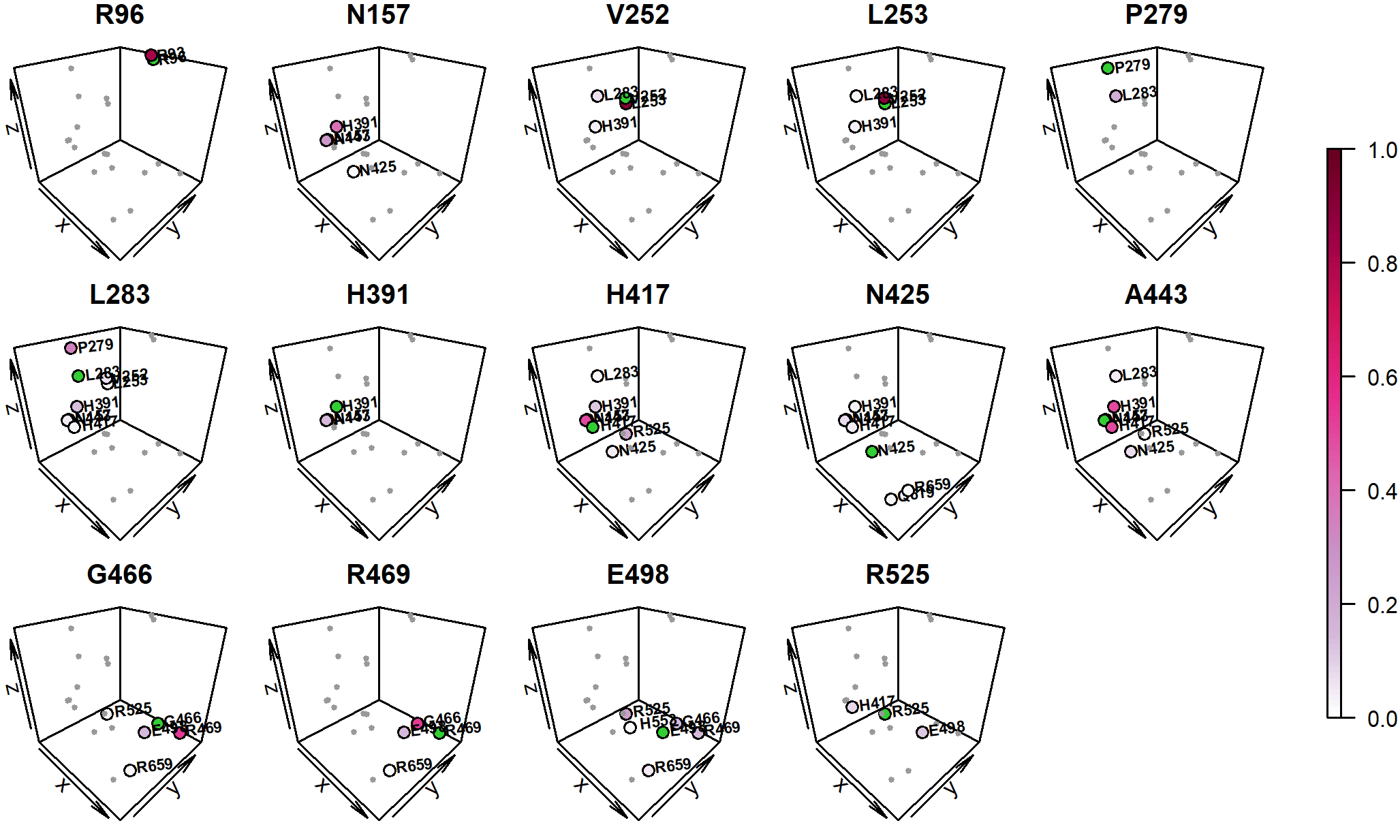

Supplement: S7 Fig — Information-borrowing map shows the amount of borrowing from neighboring variants for the chosen best c value for the local kernel test of association between the rare variants in PCSK9 and LDL. (TIFF) [file pcbi.1006722.s007.tiff]
