# Supplementary material for: Identifying individual risk rare variants using protein structure guided local tests (POINT)
Source: PLoS Comput Biol. 2019 Feb 19;15(2):e1006722. doi: 10.1371/journal.pcbi.1006722 (PMC6396946; doi:10.1371/journal.pcbi.1006722)
Supplement: S1 Table — Minor allele frequency and protein coordinate information for the rare variants in PLA2G7. The 3D coordinates are obtained from PDB entry 3F96. (PDF) [file pcbi.1006722.s008.pdf]

Table S1. *PLA2G7* rare variant summary information. Minor allele frequency and protein coordinate information for the rare variants in *PLA2G7*. The 3D coordinates are obtained from PDB entry 3F96.

**AA**	**MAF**	**3D Coordinate**		
		**x**	**y**	**z**
D69	0.0050	17.414	-13.564	24.841
R82	0.0200	18.644	-9.829	27.377
F110	0.0095	29.193	4.817	18.534
D181	0.0110	16.681	2.173	14.515
T187	0.0060	21.395	-5.889	15.509
K191	0.0045	17.951	-13.307	5.524
D200	0.0055	23.340	-17.403	9.162
S273	0.0065	21.955	9.098	24.130
V279	0.0040	9.560	6.994	26.739
L283	0.0145	3.802	7.434	26.441
G303	0.0385	8.373	16.378	17.727
A326	0.0095	18.073	22.765	23.972
M331	0.0085	12.685	18.092	29.051
