# Supplementary material for: Identifying individual risk rare variants using protein structure guided local tests (POINT)
Source: PLoS Comput Biol. 2019 Feb 19;15(2):e1006722. doi: 10.1371/journal.pcbi.1006722 (PMC6396946; doi:10.1371/journal.pcbi.1006722)
Supplement: S2 Table — Minor allele frequency and protein coordinate information for the rare variants in PCSK9. The 3D coordinates are obtained from PDB entry 4K8R. (PDF) [file pcbi.1006722.s009.pdf]

Table S2. *PCSK9* rare variant summary information. Minor allele frequency and protein coordinate information for the rare variants in *PCSK9*. The 3D coordinates are obtained from PDB entry 4K8R.

AA	SNP ID	MAF	3D Coordinate		
			x	y	z
R93	rs151193009	0.0003	57.346	-46.239	14.168
R96	rs185392267	0.0001	55.728	-42.242	11.566
N157	rs143117125	0.0001	38.018	-76.553	-23.43
V252	rs149139428	0.0001	47.715	-55.573	-7.178
L253	rs72646508	0.0003	40.829	-54.25	-10.678
P279	rs72646509	0.0001	40.775	-73.533	10.343
L283	rs72646510	0.0001	43.215	-72.831	-0.779
H391	rs146471967	0.0002	41.198	-73.649	-16.112
H417	rs143275858	0.0001	53.053	-84.877	-15.42
N425	rs28362261	0.0031	53.144	-76.191	-32.719
A443	rs28362263	0.0141	44.693	-81.951	-18.093
G466	rs72646517	0.0001	74.752	-63.34	-21.448
R469	rs141502002	0.0012	83.385	-63.452	-20.617
E498	rs145468572	0.0001	78.379	-77.199	-15.736
R525	rs140286279	0.0001	66.35	-81.468	-12.955
H553	rs28362270	0.0012	80.897	-94.859	-2.167
Q554	rs149311926	0.0003	78.241	-94.757	0.603
Q619	rs28362277	0.0024	71.961	-85.137	-38.086
R659	rs147182054	0.0003	75.068	-79.678	-34.965
