# Supplementary material for: Identifying individual risk rare variants using protein structure guided local tests (POINT)
Source: PLoS Comput Biol. 2019 Feb 19;15(2):e1006722. doi: 10.1371/journal.pcbi.1006722 (PMC6396946; doi:10.1371/journal.pcbi.1006722)
Supplement: S3 Table — Selection performance for single variant test (SVT), REBET, POINT test using local burden kernel (POINT-Burden), and POINT test using local linear kernel (POINT-Linear). The best performed methods (based on the composite F-measure) are shown in bold. (PDF) [file pcbi.1006722.s010.pdf]

Table S3. Selection performance of continuous-trait simulation with $n = 1000$ subjects. Selection performance for single variant test (SVT), REBET, POINT test using local burden kernel (POINT-Burden), and POINT test using local linear kernel (POINT-Linear). The best performed methods (based on the composite F-measure) are shown in bold and the second best are shown in *italic*.

	causal variants	(D69, R82)	(F110, S273)	(K191, D200)	(G303, A326, M331)	D69	F110	K191	(A326, M331)	(D69, R82), (G303, A326, M331)
	MAF	(0.0050, 0.0200)	(0.0095, 0.0065)	(0.0045, 0.0055)	(0.0385, 0.0095, 0.0085)	0.0050	0.0095	0.0045	(0.0095, 0.0085)	(0.0050, 0.0200), (0.0385, 0.0095, 0.0085)
TPR	SVT	0.736	0.730	0.589	0.802	0.574	0.844	0.564	0.778	0.823
	REBET	0.912	0.295	0.432	0.994	0.110	0.220	0.122	0.458	0.974
	POINT-Burden	0.971	0.844	0.800	0.930	0.500	0.831	0.546	0.906	0.971
	POINT-Linear	0.951	0.797	0.648	0.839	0.493	0.855	0.551	0.810	0.925
FDR	SVT	0.435	0.261	0.302	0.177	0.476	0.393	0.489	0.246	0.207
	REBET	0.420	0.234	0.347	0.172	0.577	0.469	0.558	0.304	0.268
	POINT-Burden	0.391	0.234	0.237	0.159	0.546	0.405	0.523	0.217	0.205
	POINT-Linear	0.373	0.233	0.276	0.163	0.538	0.387	0.505	0.232	0.195
F Measure	SVT	0.639	0.734	0.639	0.813	**0.548**	*0.706*	**0.536**	0.766	0.808
	REBET	0.709	0.426	0.520	**0.904**	0.175	0.311	0.191	0.553	0.836
	POINT-Burden	*0.749*	**0.803**	**0.781**	*0.883*	0.476	0.693	0.509	**0.840**	**0.874**
	POINT-Linear	**0.756**	*0.782*	*0.684*	0.838	*0.477*	**0.714**	*0.521*	*0.788*	*0.861*

* MAF: Minor allele frequency
