# Supplementary material for: Identifying individual risk rare variants using protein structure guided local tests (POINT)
Source: PLoS Comput Biol. 2019 Feb 19;15(2):e1006722. doi: 10.1371/journal.pcbi.1006722 (PMC6396946; doi:10.1371/journal.pcbi.1006722)
Supplement: S4 Table — Selection performance for single variant test (SVT), scan statistic (SCAN), ADA, REBET, POINT test using local burden kernel (POINT-Burden), and POINT test using local linear kernel (POINT-Linear). The best performed methods (based on the composite F-measure) are shown in bold. (PDF) [file pcbi.1006722.s011.pdf]

Table S4. Selection performance of binary trait simulation with $n = 1000$ subjects. Selection performance for single variant test (SVT), scan statistic (SCAN), ADA, REBET, POINT test using local burden kernel (POINT-Burden), and POINT test using local linear kernel (POINT-Linear). The best performed methods (based on the composite F-measure) are shown in bold and the second best are shown in *italic*.

	causal variants	(D69, R82)	(F110, S273)	(K191, D200)	(G303, A326, M331)	D69	F110	K191	(A326, M331)	(D69, R82), (G303, A326, M331)
	MAF	(0.0050, 0.0200)	(0.0095, 0.0065)	(0.0045, 0.0055)	(0.0385, 0.0095, 0.0085)	0.0050	0.0095	0.0045	(0.0095, 0.0085)	(0.0050, 0.0200), (0.0385, 0.0095, 0.0085)
TPR	SVT	0.705	0.722	0.636	0.822	0.552	0.810	0.540	0.789	0.814
	SCAN	0.767	0.290	0.560	0.899	0.154	0.402	0.236	0.707	0.398
	ADA	0.795	0.616	0.453	0.841	0.180	0.256	0.128	0.623	0.932
	REBET	0.844	0.179	0.300	0.998	0.064	0.146	0.044	0.356	0.976
	POINT-Burden	0.965	0.825	0.805	0.910	0.484	0.796	0.554	0.877	0.954
	POINT-Linear	0.934	0.758	0.651	0.827	0.516	0.832	0.527	0.789	0.903
FDR	SVT	0.465	0.267	0.274	0.169	0.504	0.406	0.504	0.244	0.215
	SCAN	0.492	0.477	0.296	0.094	0.516	0.478	0.560	0.081	0.740
	ADA	0.620	0.561	0.598	0.421	0.791	0.779	0.812	0.561	0.325
	REBET	0.446	0.260	0.398	0.185	0.644	0.538	0.703	0.380	0.284
	POINT-Burden	0.394	0.239	0.219	0.156	0.545	0.409	0.488	0.219	0.203
	POINT-Linear	0.397	0.262	0.297	0.177	0.521	0.405	0.518	0.250	0.197
F Measure	SVT	0.609	0.727	*0.678*	0.826	**0.522**	*0.685*	*0.517*	0.772	0.799
	SCAN	0.611	0.373	0.624	**0.902**	0.234	0.454	0.307	*0.799*	0.315
	ADA	0.515	0.513	0.426	0.686	0.193	0.237	0.152	0.515	0.783
	REBET	0.669	0.288	0.401	*0.898*	0.109	0.222	0.077	0.452	0.826
	POINT-Burden	**0.745**	**0.792**	**0.793**	0.876	0.469	0.678	**0.532**	**0.826**	**0.868**
	POINT-Linear	*0.733*	*0.748*	0.676	0.825	*0.497*	**0.694**	0.503	0.769	*0.850*
