# Supplementary material for: Identifying individual risk rare variants using protein structure guided local tests (POINT)
Source: PLoS Comput Biol. 2019 Feb 19;15(2):e1006722. doi: 10.1371/journal.pcbi.1006722 (PMC6396946; doi:10.1371/journal.pcbi.1006722)
Supplement: S1 Appendix — (PDF) [file pcbi.1006722.s012.pdf]

S1 Appendix.

Resampling Approach to Obtain the P-value of the Localized Test for Variant m.

1. Expressing the score test statistic $T_{m,c}$ in terms of the influence function.

Recall that the local kernel score test statistic in POINT for variant m with a fixed c is

$$T_{m,c} = \frac{1}{n}(\hat{\epsilon}_1, ..., \hat{\epsilon}_n)^T K_{m,c}(\hat{\epsilon}_1, ..., \hat{\epsilon}_n),$$

where $\hat{\epsilon}_i = Y_i - g^{-1}(X_i^T \hat{\beta})$ is the fitted residual from the KM model under the null hypothesis of no genetic effect. Let $K_{m,c} = Z_{m,c} Z_{m,c}^T$ and $\left[Z_{m,c}^{(i)}\right]^T$ be the row vector of $Z_{m,c}$. Then

$$T_{m,c} = \frac{1}{n}(\hat{\epsilon}_1, ..., \hat{\epsilon}_n) Z_{m,c} Z_{m,c}^T (\hat{\epsilon}_1, ..., \hat{\epsilon}_n)^T = \left[\frac{1}{\sqrt{n}} \sum_{i=1}^n Z_{m,c}^{(i)} \hat{\epsilon}_i\right]^T \left[\frac{1}{\sqrt{n}} \sum_{i=1}^n Z_{m,c}^{(i)} \hat{\epsilon}_i\right]$$

By Taylor expansion around the true value of β under the null, denoted by β_*, we have

$$\frac{1}{\sqrt{n}} \sum_{i=1}^n Z_{m,c}^{(i)} \hat{\epsilon}_i = \frac{1}{\sqrt{n}} \sum_{i=1}^n Z_{m,c}^{(i)} \epsilon_i + \frac{1}{\sqrt{n}} \sum_{i=1}^n Z_{m,c}^{(i)} \frac{\partial \epsilon_i}{\partial \beta^T} \left(\hat{\beta} - \beta_*\right) + o_p(1) \qquad (1)$$

where $\epsilon_i = Y_i - g^{-1}(X_i^T \beta_*)$.

For quantitative traits, Equation (1) becomes

$$\begin{aligned}
\frac{1}{\sqrt{n}} \sum_{i=1}^n Z_{m,c}^{(i)} \hat{\epsilon}_i &= \frac{1}{\sqrt{n}} \sum_{i=1}^n Z_{m,c}^{(i)} \epsilon_i - \frac{1}{n} \sum_{i=1}^n Z_{m,c}^{(i)} X_i^T \sqrt{n}(\hat{\beta} - \beta_*) + o_p(1) \\
&= \frac{1}{\sqrt{n}} \sum_{i=1}^n Z_{m,c}^{(i)} \epsilon_i - \left(\frac{1}{n} \sum_{i=1}^n Z_{m,c}^{(i)} X_i^T\right) \left(\frac{1}{n} \sum_{i=1}^n X_i X_i^T\right)^{-1} \times \\
&\qquad \frac{1}{\sqrt{n}} \sum_{i=1}^n X_i (y_i - X_i^T \beta_*) + o_p(1) \\
&= \frac{1}{\sqrt{n}} \sum_{i=1}^n \left\{Z_{m,c}^{(i)} - A_2 A_1^{-1} X_i\right\} \epsilon_i + o_p(1) \\
&\equiv \frac{1}{\sqrt{n}} \sum_{i=1}^n \psi_i + o_p(1), \qquad (2)
\end{aligned}$$

where $A_1 = E(X_i X_i^T)$ and $A_2 = E(Z_{m,c}^{(i)} X_i^T)$. Here $\psi_i = \left\{Z_{m,c}^{(i)} - A_2 A_1^{-1} X_i\right\} \epsilon_i$ is an influence function that can be consistently estimated by $\hat{\psi}_i = \left\{Z_{m,c}^{(i)} - \hat{A}_2 \hat{A}_1^{-1} X_i\right\} \epsilon_i$,

where

$$\hat{A}_1 = \frac{1}{n}\sum_{i=1}^{n} X_i X_i^T \qquad \text{and} \qquad \hat{A}_2 = \frac{1}{n}\sum_{i=1}^{n} Z_{m,c}^{(i)} X_i^T.$$

Similarly, for binary traits, we have

$$\begin{aligned}\frac{1}{\sqrt{n}}\sum_{i=1}^{n} Z_{m,c}^{(i)}\hat{\epsilon}_i &= \frac{1}{\sqrt{n}}\sum_{i=1}^{n} Z_{m,c}^{(i)}\epsilon_i - \frac{1}{n}\sum_{i=1}^{n} Z_{m,c}^{(i)}\left(\frac{e^{X_i^T\beta_*}}{(1+e^{X_i^T\beta_*})^2}X_i^T\right)\sqrt{n}\left(\hat{\beta}-\beta_*\right) + o_p(1)\\ &= \frac{1}{\sqrt{n}}\sum_{i=1}^{n}\left\{Z_{m,c}^{(i)} - A_2A_1^{-1}X_i\right\}\epsilon_i + o_p(1) \equiv \frac{1}{\sqrt{n}}\sum_{i=1}^{n}\psi_i + o_p(1),\end{aligned} \tag{3}$$

where $A_1 = E\left(\frac{e^{X_i^T\beta}}{(1+e^{X_i^T\beta})^2}X_iX_i^T\right)$ and $A_2 = E\left(Z_{m,c}^{(i)}\frac{e^{X_i^T\beta}}{(1+e^{X_i^T\beta})^2}X_i^T\right)$. In addition, A_1 and A_2 can be consistently estimated respectively by

$$\hat{A}_1 = \frac{1}{n}\sum_{i=1}^{n}\frac{e^{X_i^T\hat{\beta}}}{(1+e^{X_i^T\hat{\beta}})^2}X_iX_i^T \qquad \text{and} \qquad \hat{A}_2 = \frac{1}{n}\sum_{i=1}^{n} Z_{m,c}^{(i)}\frac{e^{X_i^T\hat{\beta}}}{(1+e^{X_i^T\hat{\beta}})^2}X_i^T.$$

2. Details of the weighted chi-square distribution of $T_{m,c}$

By the central limit theorem, we have $\frac{1}{\sqrt{n}}\sum_{i=1}^{n} Z_{m,c}^{(i)}\hat{\epsilon}_i \xrightarrow{d} N(0,\Sigma_{m,c})$, where

$$\Sigma_{m,c} = E(\psi_i\psi_i^T) = E\left[\left\{Z_{m,c}^{(i)} - A_2A_1^{-1}X_i\right\}^{\bigotimes 2}\epsilon_i^2\right], \tag{4}$$

which can be consistently estimated by

$$\hat{\Sigma}_{m,c} = \frac{1}{n}\sum_{i}^{n}\left\{Z_{m,c}^{(i)} - \hat{A}_2\hat{A}_1^{-1}X_i\right\}^{\bigotimes 2}\hat{\epsilon}_i^2, \tag{5}$$

where $a^{\bigotimes 2} = aa^T$. Thus,

$$T_{m,c} = \left[\frac{1}{\sqrt{n}}\sum_{i=1}^{n} Z_{m,c}^{(i)}\hat{\epsilon}_i\right]^T\left[\frac{1}{\sqrt{n}}\sum_{i=1}^{n} Z_{m,c}^{(i)}\hat{\epsilon}_i\right] \xrightarrow{d} \sum_{d}\xi_d\chi_{1,d}^2$$

where ξ_d are the nonzero eigenvalues of $\Sigma_{m,c}$ and can be estimated by $\hat{\xi}_d$, the nonzero eigenvalues of $\hat{\Sigma}_{m,c}$.

3. The resampling algorithm to obtain the p-value of minP over a grid of c values.

Recall that for variant m, given a grid of c's, $c = c_1, ..., c_L$, we adaptively find the optimal c by choosing the c that yields minP= $\min\{p_{m,c_1}, ..., p_{m,c_L}\}$. Here we describe the approach to obtain the p-value of minP (denoted by p_m^*) for

ranking the variants. Specifically, given that $T_{m,c} = \left(\frac{1}{\sqrt{n}}\sum_{i=1}^{n}\psi_i\right)^T\left(\frac{1}{\sqrt{n}}\sum_{i=1}^{n}\psi_i\right) + o_p(1)$ and ψ_i's can be consistently estimated by $\hat{\psi}_i$'s, we can obtain the p-value of the minP of variant m by perturbing the estimated influence functions $\hat{\psi}_i$'s. That is, we generate B random vectors of length n: $O_1, ..., O_B$ from a standard Normal distribution and create B perturbed test statistics of variant m for each c value:

$$T_{m,c}^{(b)} = \left(\frac{1}{\sqrt{n}}\sum_{i=1}^{n} O_{ib}\hat{\psi}_i\right)^T \left(\frac{1}{\sqrt{n}}\sum_{i=1}^{n} O_{ib}\hat{\psi}_i\right), b = 1, ..., B$$

These $T_{m,c}^{(b)}$'s have to be converted to p-values, denoted as $p_{m,c}^{(b)}$'s, e.g., using Davies method so to be comparable across different c values. We can then calculate the bth perturbed minP statistic over different c's, i.e., $\min P_m^{(b)} = \min_c\{p_{m,c}^{(b)}\}$, $b = 1, ..., B$. Finally, our minP p-value for variant m is $p_m^* = \frac{1}{B}\sum_{b=1}^{B} I(\min P_m^{(b)} < \min P_m)$, where $I(\cdot)$ is the indicator function. These p-values can be used to rank and select promising variants, e.g., to select the top J variants with p_m^* less than a certain threshold.
