# Supplementary material for: Identifying individual risk rare variants using protein structure guided local tests (POINT)
Source: PLoS Comput Biol. 2019 Feb 19;15(2):e1006722. doi: 10.1371/journal.pcbi.1006722 (PMC6396946; doi:10.1371/journal.pcbi.1006722)

HDL vs. ANGPTL4

Variant ID (AA Coord)	SNP RSID	MAF	Single Variant Test (SVT) p-value	POINT-Burden		REBET	
				p-value	best c	Subregion Defintion	p-value
E190	rs77938377	0.0022	0.6232	**<0.001**	0.5	1	**2.39E-06**
G223	rs148185134	0.0130	**<0.001**	**<0.001**	0		
R278	rs35061979	0.0001	0.3822	0.532	0		
V308	rs139998264	0.0001	0.8219	0.247	0.4	2	**0.040**
G321	rs143596863	0.0011	0.2002	0.238	0.5		
Q331	rs146875081	0.0002	0.1056	**0.030**	0.5	3	
R336	rs140744493	0.0049	0.0670	0.079	0.5		
R365	rs138460722	0.0001	0.4878	0.497	0		

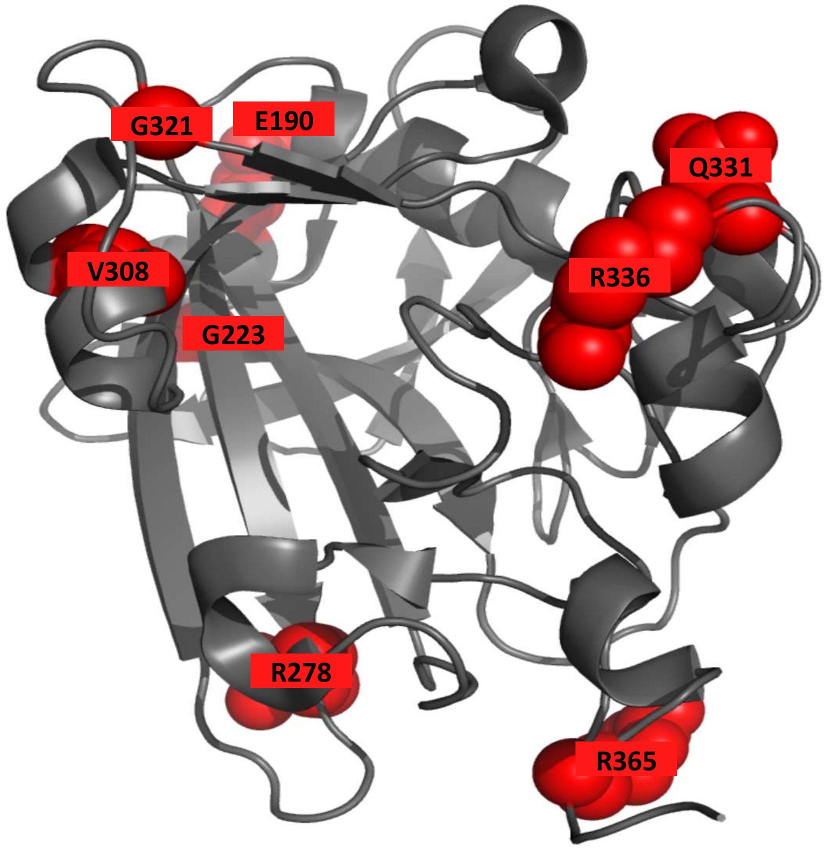

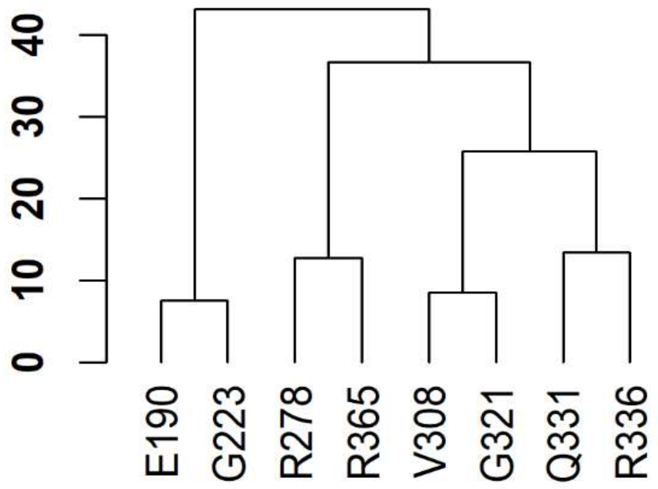

Supplement: S2 Appendix — (PDF) [file pcbi.1006722.s013.pdf]
