# Supplementary material for: Identifying individual risk rare variants using protein structure guided local tests (POINT)
Source: PLoS Comput Biol. 2019 Feb 19;15(2):e1006722. doi: 10.1371/journal.pcbi.1006722 (PMC6396946; doi:10.1371/journal.pcbi.1006722)

HDL vs. CETP

Variant ID (AA Coord)	SNP RSID	MAF	Single Variant Test (SVT) p-value	POINT-Burden		REBET p-value	
				p-value	best c	Subregion Definition	p-value
T61	rs142464301	0.0133	**<0.001**	**<0.001**	0	1	**2.70E-08**
Q128	rs140518082	0.0003	0.3983	**<0.001**	0.5		
R154	rs34716057	0.0003	0.8378	0.065	0.5		
Q182	rs142750310	0.0049	0.1204	0.025	0.5	2	not sig
S221	rs201438792	0.0001	0.3170	0.483	0		
G251	rs144460063	0.0001	0.4410	0.602	0.2	3	not sig
L262	rs150475423	0.0001	0.2950	0.338	0.3		
R299	rs142459781	0.0001	0.2206	0.418	0		
E314	rs140547417	0.0001	0.7670	0.924	0.3		
G331	rs5881	0.0008	0.8625	0.930	0.5	4	not sig
V340	rs141310739	0.0002	0.4937	0.752	0		
V385	rs34855278	0.0001	0.4697	0.607	0.2		
T415	rs190964678	0.0017	0.2931	0.403	0		

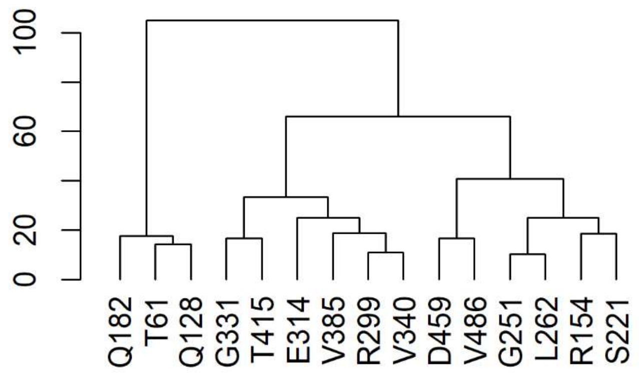

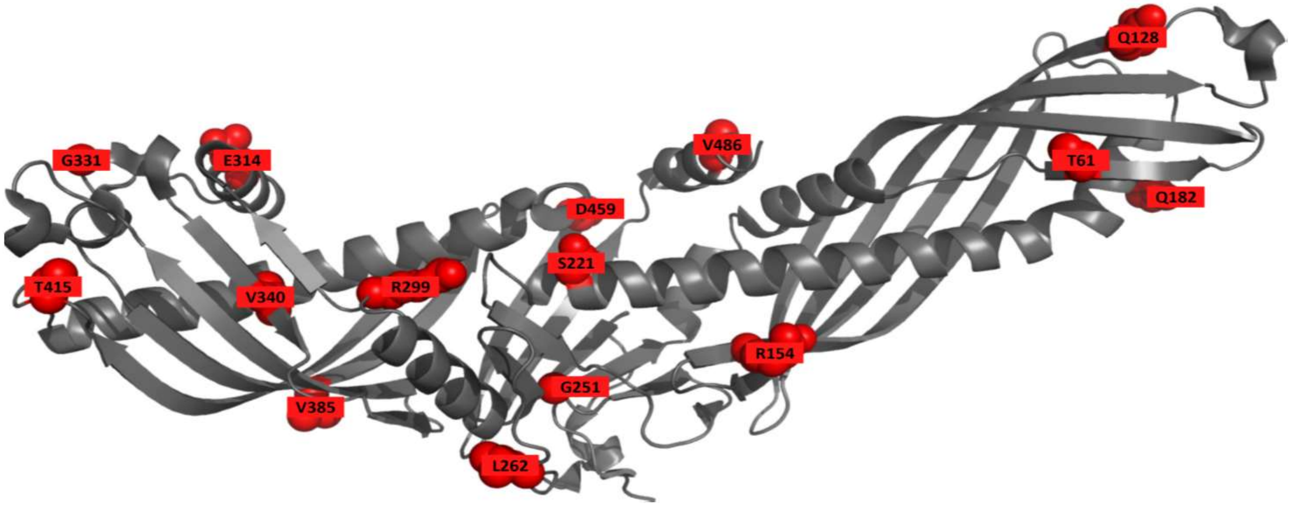

Supplement: S3 Appendix — (PDF) [file pcbi.1006722.s014.pdf]
